# Injured bone-triggered osteokines secretion promotes diabetic wound healing

**DOI:** 10.1038/s41413-025-00454-9

**Published:** 2025-10-02

**Authors:** Tong Shen, Kai Dai, Shuang Zhang, Jing Wang, Changsheng Liu

**Affiliations:** 1 State Key Laboratory of Bioreactor Engineering, East China University of Science and Technology, Shanghai, PR China; 2 Engineering Research Center for Biomedical Materials of the Ministry of Education, East China University of Science and Technology, Shanghai, PR China; 3 Key Laboratory for Ultrafine Materials of the Ministry of Education, East China University of Science and Technology, Shanghai, PR China; 4 Frontiers Science Center for Materiobiology and Dynamic Chemistry, East China University of Science and Technology, Shanghai, PR China

**Keywords:** Bone, Neurophysiology

## Abstract

The treatment of severe diabetic foot remains a clinical challenge. While it is established that bone can exert systemic effects through the secretion of osteokines on other organs, whether this endocrine function can be harnessed to promote diabetic wound healing remains unexplored. Here, we investigate the impact of a bone injury strategy on diabetic wound healing, leveraging the body’s innate regenerative capacity to stimulate osteokine release and influence remote skin wound repair. This study demonstrates that the tibial defect significantly accelerates ipsilateral diabetic foot skin wound healing. Mechanistically, we identify osteokines, platelet-derived growth factor-BB (PDGF-BB), as the key to initiating this process. Bone defect triggers a substantial release of PDGF-BB, which reaches the skin wound site via peripheral circulation. At the skin wound site, PDGF-BB mediates the secretion of keratinocyte growth factor (KGF) from fibroblasts via the PDGFRβ signaling pathway, thereby promoting the rapid re-epithelialization of epidermal cells through a paracrine pathway. Additionally, elevated PDGF-BB levels enhance the regeneration of CD31^hi^ Emcn^hi^ blood vessels within the wound. Importantly, we demonstrate the therapeutic potential of osteokines by showing that a collagen hydrogel loaded with osteokines promotes wound healing in diabetic mice. Our findings reveal a clear link between bone and skin wound healing, providing a therapeutic inspiration for chronic wounds that are difficult to treat locally.

## Introduction

Diabetic foot ulcers (DFUs) are a common and destructive complication of diabetes and one of the most common types of chronic wounds. The typical characteristics of DFUs include insufficient wound epithelialization, difficulty in vascular regeneration, chronic inflammation, abnormal collagen deposition, etc.^1^ At present, the treatment of diabetic foot mainly includes local wound debridement, negative pressure closed drainage, and local dressing treatment.^2–4^ However, diabetic foot patients are often accompanied by lower extremity vascular and neuropathy, resulting in large and deep foot ulcers, that make local wound treatment difficult to achieve the best therapeutic effect and may eventually lead to a high incidence of amputation.^5,6^ Therefore, it is urgent to develop a new treatment method to activate the body’s own regenerative ability and achieve effective repair of diabetic foot.

With the gradual deepening of cross-organ research, we have realized that there is a close relationship between various solid organs of the human body. In particular, bone, as an endocrine organ, is involved in regulating the homeostasis of other non-bone organs by releasing bone-derived factors.^7,8^ Such as myeloid-derived growth factor, inhibits endothelial cell damage caused by inflammation, thereby inhibiting the progression of atherosclerosis.^9^ Bone can also secrete osteocalcin and lipocalin-2, which affect brain function.^10^ The skin, as the largest organ of the human body, serves as an important receptor for external stimuli and regulates other organs through paracrine and other effects. It is already known that skin inflammation in psoriasis patients will lead to increased levels of IL-17A in serum, specifically inhibiting bone formation of osteoblasts and resulting in osteoporosis.^11^ Other studies have shown that in the process of normal aging, the secretion of cystatin-A in aging epidermal cells is reduced, which is an important reason for affecting osteoporosis.^12^ These evidences suggest that there is a strong connection between bone and skin.

During the process of bone regeneration, the levels of growth factors and chemokines such as granulocyte-macrophage colony-stimulating factor, vascular endothelial growth factor (VEGF), Angiopoietin-1, and interleukin-6 (IL-6) in the bone marrow microenvironment increased, while serum factor levels also change correspondingly.^13,14^ A number of clinical studies have shown that changing the bone microenvironment through external stimulation can reconstruct the lower limb blood flow of diabetic patients and achieve the purpose of treating diabetic foot.^15–17^ The rat experiment also confirmed that tibial cortex transverse transport accelerates wound healing via enhanced angiogenesis and immunomodulation.^18^ Inspired by these studies, we speculate that it may be possible to promote diabetic foot repair by initiating endogenous bone regeneration. However, the underlying mechanism through which bone microenvironment alterations influence skin wound healing remains to be elucidated. Understanding how bone promotes wound healing not only provides compelling evidence for bone-mediated cross-organ regulation of skin regeneration but also contributes to the development of potential therapies for chronic wounds.

Here we demonstrate that tibial bone defects (BD) trigger an unexpected acceleration of wound healing in a diabetic mouse model. Specifically, we observed enhanced re-epithelialization and revascularization of ipsilateral diabetic foot wounds following tibial injury. Mechanistically, plasma analysis revealed that factors released from the bone injury promote cellular crosstalk between fibroblasts and keratinocytes. We identified PDGF-BB as a key molecular mediator orchestrating the bone-skin healing axis. To validate the therapeutic potential of these findings, we developed bone-derived factor-loaded hydrogels, which significantly enhanced dorsal skin wound healing in diabetic mice. These results uncover a previously unrecognized bone-skin healing circuit and establish bone-derived factors as promising therapeutic agents for diabetic wound treatment.

## Results

### Bone defect accelerates skin wound healing in diabetic mice

**Fig. 1:**
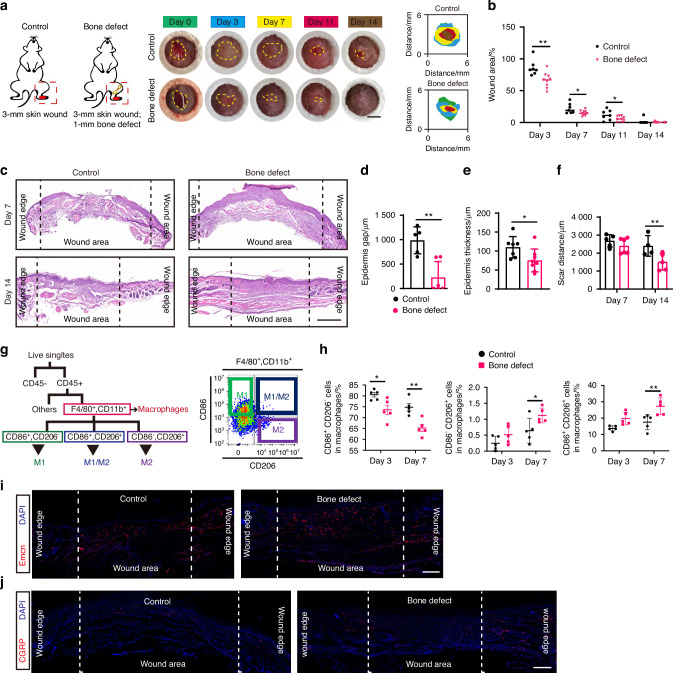
Bone defect induces more effective healing of diabetic foot wounds. **a** Surgical diagram and representative images of foot healing in diabetic mice and bone defect diabetic mice. The yellow dashed line represents the remaining wound contour. Scale bars, 2 mm. **b** Quantification of the percent of foot wound residual area at different times in control and bone defect groups. (*n* = 7 and 9, respectively). **c** Representative H&E staining of foot wound tissue on days 7 and 14. Scale bars, 500 μm. **d** Quantification of epidermis gap on day 7, **e** epidermal thickness on day 14, and **f** scar distance on days 7 and 14 according to H&E staining images (*n* = 5). **g** Schematic diagram of gating strategies for macrophage subpopulations analysis by flow cytometry. **h** Quantification of the percentage of CD86^+^CD206^−^ macrophages, CD86^+^CD206^+^, and CD86^−^CD206^+^ macrophages in wound tissues on days 3 and 7. Sagittal sections of day 14 wound tissue immunolabeled for Emcn (red) (**i**) and CGRP (red) (**j**), cell nucleus for DAPI (blue). Scale bar, 200 μm. Data are presented as mean ± SD and statistical significance was analyzed via two-way ANOVA with Tukey’s multiple comparison test for (**b**), (**f**), (**h**), unpaired two-tailed Student’s *t* test for (**d**, **e**). *P* value: *P < 0.05, **P < 0.01

We initially employed streptozotocin (STZ)-induced diabetic mice as our experimental animal model, which demonstrated characteristic diabetic phenotypes (Fig. S1). To elucidate the impact of BD on skin wound healing, we established a BD group by creating a unilateral tibial defect in diabetic mice, accompanied by a full-thickness skin wound on the ipsilateral foot (Fig. S2). The control group only had foot skin wound. The wound areas in different groups were measured and photographed on day 0, 3, 7, 11, and 14. As shown in Fig. 1a, b, BD treatment notably accelerated the closure of diabetic wounds, especially on day 3 post-injury, where the average residual wound area was 66.21%, compared to 86.14% in the control group. The period from day 3 to day 7 represented an accelerated phase of wound healing, during which both groups showed wound closure. While the difference in residual wound area gradually narrowed, a statistically significant difference between the groups persisted.

Further Hematoxylin and Eosin (H&E) staining analysis showed that by day 7, skin tissues in the BD group had achieved preliminary re-epithelialization, despite visible scarring in wound appearance photographs, whereas the control group still exhibited a substantial epidermal gap (Fig. 1c, d). By day 14, wound tissues in both groups had completed re-epithelialization, and epidermal and dermal tissues began to remodel. The granulation tissue in the BD group was thinner compared to the control group, gradually returning to normal epidermal thickness. Denser collagen deposition and smaller scar distance were also found in BD group wound tissue. (Fig. 1c, e, f).

Chronic inflammation is a typical characteristic of diabetic wounds, where abnormal macrophage polarization is a major factor causing chronic inflammation.^19,20^ Therefore, we also examined whether BDs would affect the inflammatory response of diabetic foot wounds. To verify this hypothesis, we used flow cytometry to analyze macrophage polarization on days 3 and 7, using CD86 and CD206 as markers for pro-inflammatory (M1) and anti-inflammatory (M2) phenotype macrophages, respectively. The results showed that after BD treatment, the proportion of CD86^+^CD206^−^ M1 macrophages in diabetic wounds significantly decreased, and the proportion of CD86^+^CD206^+^ macrophages and CD86^−^CD206^+^ M2 macrophages significantly increased on day 7 (Figs. 1g, h and S4). These results suggested BD-activated M2 macrophage polarization in the ipsilateral diabetic foot wound tissue, thereby inhibiting chronic inflammation.

Vascular regeneration plays a critical role in maintaining tissue survival by providing oxygen and nutrients to the tissues. Therefore, we also analyzed the vascularization in the wound bed on day 14. The results of Endomucin (Emcn) immunofluorescence staining showed that there were more newly formed Emcn^+^ vessels distributed in the wound bed of the BD group compared to the control group (Fig. 1i). Skin tissue is highly innervated, and it has been proven that various neuropeptides participate in wound repair, such as calcitonin gene-related peptide (CGRP), neuropeptide Y.^21^ However, peripheral neuropathy is a common complication in diabetic patients, making nerve recovery an important indicator for evaluating diabetic wound repair.^22,23^ The results of immunofluorescence staining showed that there were more CGRP^+^ sensory nerves distributed around the hair follicles and epidermal areas at the edge of the wound in the BD group. There were also strong CGRP^+^ signals distributed in the wound bed area (Fig. 1j).

The above results indicate that BD enhances ipsilateral diabetic foot wound healing, promoting early wound closure, granulation tissue remodeling, vascular regeneration, and sensory nerve recovery.

### Bone defect activates epidermal re-epithelialization in diabetic mice

**Fig. 2:**
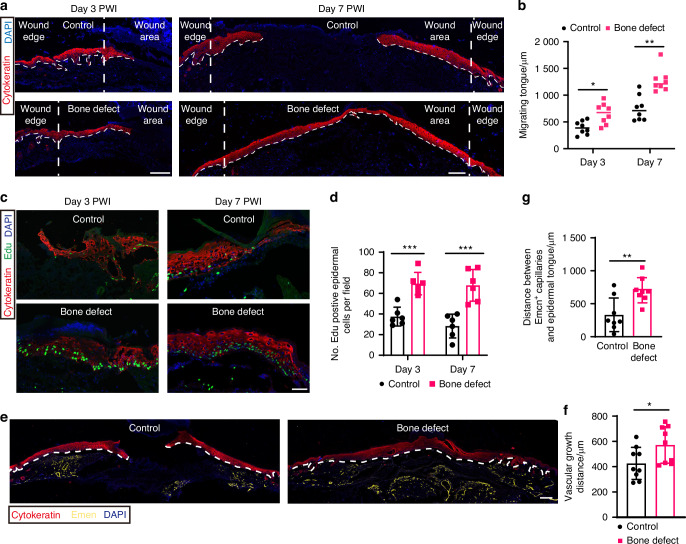
Bone defect promotes epidermal migration and proliferation of diabetic wound. **a** Sagittal sections of days 3 and 7 wound tissue immunolabeled for cytokeratin (red) and DAPI (blue). Scale bar, 200 μm. **b** Quantitative statistics of the migration distance of epidermal tongue from wound edge (*n* = 8). **c** Sagittal sections of days 3 and 7 wound tissue immunolabeled for cytokeratin (red), and labeled with 5-ethynyl-2′-deoxyuridine (EdU) (green) (proliferation), cell nucleus for DAPI (blue). Scale bar, 100 μm. **d** Quantitative statistics of the number of Edu^+^ epidermal cells in each visual field (*n* = 6). **e** Fluorescence microscopy of sagittal sections of day 7 wound tissue immunolabeled for cytokeratin (red) and Emcn (yellow), cell nucleus for DAPI (blue). Scale bar, 200 μm. **f** Quantitative statistics of the migration distance of Emcn^+^ blood vessel from wound edge (*n* = 8). **g** Quantification of the distance from the migrating front of the epidermal tongue to the nearest Emcn^+^ vessel was performed based on immunofluorescence staining photographs of tissue sections (*n* = 8). Data are presented as mean ± SD and statistical significance was analyzed via two-way ANOVA with Tukey’s multiple comparison test for (**b**, **d**), unpaired two-tailed Student’s *t* test for (**f**, **g**). *P* value: *P < 0.05, **P < 0.01, ***P < 0.001

During the skin repair process, epidermal cells proliferate, migrate, and differentiate to complete the re-epithelialization of the wound, restoring barrier function and preventing wound infection.^24,25^ We further analyzed epidermal epithelialization following BD treatment, using cytokeratin to mark epidermal cells. We found that the BD treatment induced the rapid migration of epidermis from the edge of the skin wound towards the wound center. The average migration distance was significantly greater in the BD group than in the control group. By day 7, the migrating epidermal cells had fused and re-epithelialization was completed in BD group, whereas it remained incomplete in the control group (Fig. 2a, b).

We also found that the basal layer of epidermal cells in diabetic wounds displayed marked proliferation defects. BD treatment could activate the proliferation of basal layer epidermal cells, thereby accelerating the rapid re-epithelialization of the wound (Fig. 2c, d). Interestingly, we found that during the healing process of diabetic foot wounds, the rate of re-epithelialization of the epidermis was faster than the rate of vascular migration. Although BD treatment could increase the migration of blood vessels towards the wound bed, the distance of epidermal migration is significantly greater than that of vascular migration. The average distance between the migrating epidermis and wound capillaries in the BD group was 704.8 μm, while that of the control group was 332.3 μm (Fig. 2e–g). Based on these results, we propose that the healing of diabetic wounds induced by BD treatment can be attributed to rapid re-epithelialization, which provides a complete barrier for the wound, thereby accelerating the regeneration of internal dermal tissue.

To exclude the potential confounding effects of soft tissue damage, including muscle injury, incurred during BD surgery on foot wound healing, we compared the wound healing outcomes between a sham surgery group (Fig. S2b) and the control group. Wound closure results demonstrated no significant difference in residual wound areas between the control and sham groups (Fig. S3a, b). Furthermore, both H&E staining and immunofluorescence analysis revealed that the wounds in these two groups had not fully re-epithelialized by day 7 (Fig. S3c, d, g). The thickness of the newly formed epidermis and the scar distance showed no significant differences (Fig. S3e, f). In contrast, non-diabetic healthy mice (WT) exhibited superior wound healing rates and quality compared to their diabetic counterparts (Control and sham groups) (Fig. S3). These findings suggest that the minimal soft tissue damage incurred during the creation of the BD model does not significantly contribute to foot wound healing.

### Plasma from bone defect diabetic mice promotes fibroblast mediated re-epithelialization via the KGF induced FGFR-2β signaling pathway

**Fig. 3:**
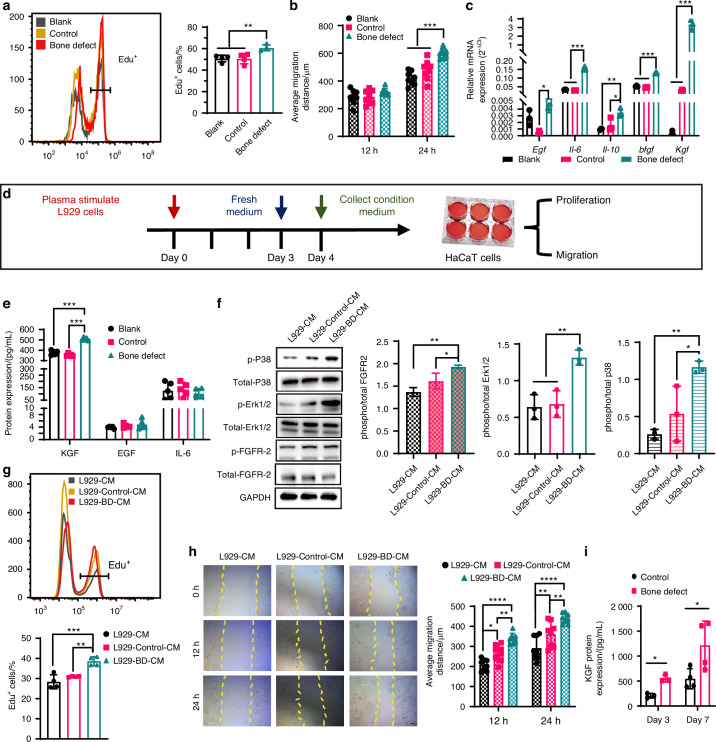
Plasma from bone defect diabetic mice promotes epidermal cell proliferation and migration by enhancing fibroblasts to secrete KGF. **a** Quantitative statistics of the percentage of Edu^+^ L929 cells by flow cytometry. Cells were treated with 10% FBS (Blank) and 10% FBS combining 5% control diabetic mice plasma (Control) or bone defect diabetic mice plasma (Bone defect), respectively (*n* = 4) for 48 h. **b** The average migration distance of L929 cells in different groups was quantitatively analyzed according to scratch test (*n* = 8). Yellow dashed line marks scratch wound edges. **c** Relative mRNA expressions of L929 cells by qPCR (*n* = 3). Cells were treated with 10% FBS (Blank) and 10% FBS combining 5% control diabetic mice plasma (Control) or bone defect diabetic mice plasma (Bone defect), respectively for 72 h (*n* = 4). **d** Schematic illustration of the L929 cell conditional medium harvesting and culture of HaCaT cells. **e** Relative protein expressions of L929 cell conditional medium by Elisa (*n* = 5). **f** Western blot analysis and the relative level of phosphorylated FGFR-2, Erk1/2, and P38 in HaCaT cells after treating with the indicated CM for 20 min (*n* = 3). **g** Quantitative statistics of the percentage of Edu^+^ HaCaT cells by flow cytometry. Cells were treated with L929-CM, L929-Control-CM, and L929-BD-CM, respectively for 48 h (*n* = 4). **h** The average migration distance of HaCaT cells treated with differenced CM was quantitatively analyzed according to scratch test. The yellow dashed line marks scratch wound edges (*n* = 8). Scale bar, 200 μm. **i** KGF protein concentrations by ELISA in wound tissue on days 3 and 7 (*n* = 4). Data are presented as mean ± SD and statistical significance was analyzed via one-way ANOVA with Tukey’s multiple comparison test for (**a**, **c**, **d**, **f**, **g**), two-way ANOVA with Tukey’s multiple comparison test for (**b**, **h**, **i**). *P* value: *P < 0.05, **P < 0.01, ***P < 0.001

To further investigate the mechanism by which BD promotes re-epithelialization, we hypothesized that the process of bone injury regeneration may involve the secretion of bone-derived factors, which circulate to the skin wound site via peripheral blood and participate in wound healing. To test this hypothesis, we first analyzed the impact of plasma from diabetic mice with BDs on the proliferation and migration of epidermal cells, using plasma from diabetic mice without BD treatment as a control. The results showed that peripheral blood plasma from BDs did not significantly affect the proliferation and migration of HaCaT epidermal cells (Fig. S5a, b). However, the proliferation and migration abilities of L929 fibroblasts were significantly enhanced by culturing with plasma from the BD group (Figs. 3a, b, and S5c), while the control plasma showed no promoting effect.

Moreover, the BD plasma significantly enhanced the expression of *Egf*, *Il-6*, *Il-10*, *bFgf*, and *Kgf* genes in L929 cells (Fig. 3c). These factors are closely related to the crosstalk between fibroblasts and keratinocytes.^26^ Particularly, KGF (FGF7), a member of the FGF family, is produced by fibroblasts and other mesenchymal cells.^27^ KGF signal transduction occurs following the activation of KGFR, an alternative splicing variant of FGFR-2β, which is predominantly expressed on epithelial cells.^28^ During wound healing, KGF plays a vital role in re-epithelialization by influencing the morphogenesis, proliferation, and migration of keratinocytes, and also contributes to hair follicle development.^27,29^ Consequently, we further investigated whether plasma activates the re-epithelialization of epidermal cells by mediating the paracrine pathway of fibroblasts. We harvested the conditioned medium from L929 (L929-CM), L929 stimulated by diabetic mice plasma (L929-control-CM), or plasma from diabetic mice with a BD (L929-BD-CM) to culture HaCaT cells (Fig. 3d). We detected the protein levels of EGF, IL-6, and KGF in the conditioned medium by Elisa. Although both EGF and IL-6 promote the proliferation and migration of epidermal cells, their protein levels showed no significant differences in the conditioned medium, whereas KGF protein levels increased in L929-BD-CM compared to the other two groups (Fig. 3e). Moreover, L929-BD-CM activated transduction of the KGF signaling cascade pathway in HaCaT cells, as demonstrated by increased phosphorylation of FGFR-2β and its downstream mediators Erk1/2 and p38 (Fig. 3f). The phosphorylation of EGFR was not different among all groups (Fig. S6).

As expected, we found that L929-BD-CM significantly promotes the proliferation of HaCaT cells, while L929-control-CM has no significant effect on HaCaT cell proliferation compared to L929-CM (Fig. 3g). Both L929-control-CM and L929-BD-CM have a noticeable promoting effect on HaCaT cell migration relative to L929-CM, with L929-BD-CM exhibiting a more pronounced migratory capability (Fig. 3h). We also found that BD treatment can significantly increase the protein level of KGF in the foot wound of diabetic mice on days 3 and 7 (Fig. 3i). In view of the fact that macrophages also can promote the proliferation and migration of epidermal cells through the secretion of growth factors,^30^ we analyzed the effect of the conditioned culture from bone marrow derived macrophages (BMDMs) treated with different plasma on epidermal cells. The results showed that different BMDMS-CM had no significant effect on both the proliferation and migration of epidermal cells (Fig. S7).

In conclusion, we proposed that bone-derived factors secreted following BD acted on the wound site through the peripheral blood circulation, thereby inducing fibroblasts to secrete KGF and activate downstream signals in epidermal cells for promoting wound re-epithelialization.

### Bone defect-triggered PDGF-BB is a potential pro-regenerative factor

**Fig. 4:**
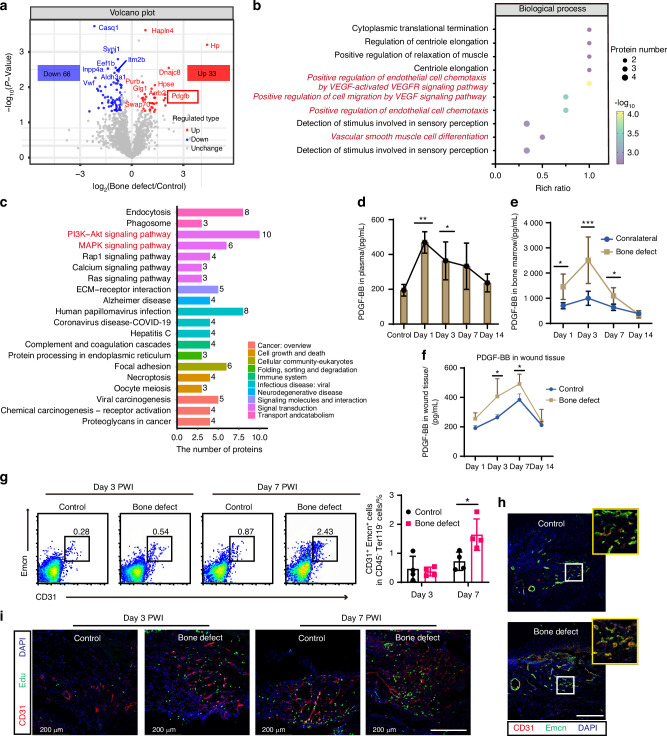
The plasma of bone defect contains PDGF-BB and is conducive to vascular regeneration of CD31^hi^ Emcn^hi^ in diabetic wounds. **a** Volcano plot of proteomics analysis of differentially expressed protein, comparing control diabetic mice plasma and bone defect diabetic mice plasma (*n* = 3). Red dots represent upregulated genes, and blue dots represent downregulated genes; adjusted *P* ≤ 0.05. **b** Top 10 GO biological process enrichment analysis for the upregulated differentially proteins (Bone defect versus Control). The size of the dots indicates the number of proteins associated with indicated GO terms and the color of the dots indicates the adjusted log *P* values calculated by one-sided Fisher’s exact test with Benjamini-Hochberg correction. **c** Top 20 KEGG pathways enriched in differentially proteins (Bone defect versus Control). The size of the dots indicates the number of genes associated with indicated KEGG terms. PDGF-BB protein expression in the plasma of the control group and the bone defect group at different time points following bone defect (**d**), in the bone defect bone marrow and contralateral uninjured bone marrow (**e**), and in the wound tissue (**f**) was measured by Elisa. **g** Representative flow cytometry plots and quantification of CD31^hi^Emcn^hi^ endothelial cells from different wound tissue at days 3 and 7 (*n* = 4). **h** Sagittal sections of foot wound tissue immunolabeled for CD31 (red), and Emcn (green), cell nucleus for DAPI (blue) at days 3 and 7. Scale bar, 200 μm. **i** Sagittal sections of foot wound tissue immunolabeled for Emcn (red), and labeled with 5-ethynyl-2′-deoxyuridine (EdU) (green, proliferation), cell nucleus for DAPI (blue) at days 3 and 7. Scale bar, 200 μm. Data are presented as mean ± SD and statistical significance was analyzed via one-way ANOVA with Tukey’s multiple comparison test for (**d**), two-way ANOVA with Tukey’s multiple comparison test for (**e**, **f**, **g**). *P* value: *P < 0.05, **P < 0.01, ***P < 0.001

Next, we focused on identifying the specific protein factors in BD-derived plasma that facilitate epidermal regeneration and wound healing. To this end, we performed a proteomics analysis to screen for differentially expressed proteins in the control and BD-derived plasma. Plasma from BD diabetic mice exhibited 99 differentially expressed proteins compared to the control plasma, with 33 proteins upregulated and 66 downregulated (Figs. 4a and S8a). The upregulated differentially expressed proteins were collected to perform Gene Ontology (GO) database analysis. Differential proteins were enriched in biological processes associated with angiogenesis, including positive regulation of endothelial cell chemotaxis by VEGF-activated VEGF receptor signaling pathway, positive regulation of cell migration by VEGF signaling pathway, positive regulation of endothelial cell chemotaxis and vascular smooth muscle cell differentiation (Figs. 4b and S8b). Furthermore, the potential signaling pathways were analyzed with the Kyoto Encyclopedia of Genes and Genomes (KEGG). As depicted in Fig. 4c, differential proteins were enriched in the PI3K-Akt signaling pathway and the MAPK signaling pathway, which correlated with signal transduction.

Among the top 10 upregulated differentially expressed proteins (Fig. S8c), Haptoglobin is predominantly synthesized in the liver, whereas Dnajc8, H2ac4, and Timp3 are not conventionally classified as secretory proteins.^31–33^ Despite ranking fifth among the upregulated proteins, PDGF-BB functions as a crucial growth factor in wound repair and tissue regeneration, possessing the unique ability to be secreted by bone tissue for distant organ regulation.^34,35^ Therefore, we identified PDGF-BB as the key mediator in bone-facilitated wound healing. We used ELISA to measure the expression levels of PDGF-BB protein in the plasma, and the results were consistent with the proteomics analysis. Specifically, the levels of PDGF-BB protein in the plasma of diabetic mice peaked on day 1 after BD, followed by a gradual decline, while maintaining elevated levels on days 3 and 7, before returning to baseline levels by day 14 (Fig. 4d). Previous studies reported that PDGF-BB secreted by preosteoclasts induces angiogenesis during coupling with osteogenesis.^36^ Consistent with our observations, the levels of PDGF-BB protein in the tibia bone marrow on days 1, days 3 and 7 after BD were significantly higher than in the contralateral uninjured bone. PDGF-BB concentrations reached their maximum on day 3, then progressively decreased, eventually equalizing with levels in the contralateral uninjured bone marrow by day 14 (Fig. 4e). Based on the aforementioned results, we propose that the substantial production of PDGF-BB during the regeneration process following BD is the primary cause of the increased levels of PDGF-BB protein in peripheral blood plasma. We also found that after BD treatment, the levels of PDGF-BB in skin wounds were higher than in the control group. PDGF-BB levels began to increase gradually from day 1 post-injury, peaked on day 7. By day 14, when the skin has fully healed, levels returned to baseline, similar to the control group (Fig. 4f).

To directly evaluate whether the elevated plasma levels of PDGF-BB induced by bone injury are a factor affecting diabetic wound healing, we increased peripheral PDGF-BB levels through retro-orbital intravenous injection (Fig. S9a). The results indicated that systemic delivery of PDGF-BB promoted early closure of diabetic foot wounds (days 3 and 7) (Fig. S9b, c). H&E staining revealed that, on day 7, the epidermal gap was narrower in both the L-PDGF-BB and H-PDGF-BB groups compared to the control group, with re-epithelialization nearly complete (Fig. S9d, e). By day 14, wounds in all groups had fully healed, and no significant differences in scar distances were identified (Fig. S9d, f). Immunofluorescence staining for cytokeratin confirmed these findings, demonstrating improved re-epithelialization in PDGF-BB-treated wounds compared to untreated controls (Fig. S9g). Collectively, these findings demonstrated that systemic administration of PDGF-BB promotes diabetic wound healing, with higher PDGF-BB concentrations exerting a more pronounced beneficial effect.

Whether the size of BDs affects the secretion of PDGF-BB and wound healing capacity in diabetes is critically important for understanding the relationship between bone and wound healing. To investigate this, we designed three distinct defect sizes (0.5, 1.0, and 1.2 mm) to evaluate their effects (Fig. S10a). The results showed that bone injury increased plasma PDGF-BB levels across all groups, although this increase was modest and transient in the 0.5 mm group, returning rapidly to baseline. Interestingly, the larger 1.2 mm defect did not result in higher plasma PDGF-BB levels compared to the 1.0 mm defect on days 1 and 3. However, by day 7, PDGF-BB concentrations were elevated in the 1.2 mm group, indicating that larger BDs do not enhance early PDGF-BB secretion but sustain elevated PDGF-BB levels for a longer duration (Fig. S10b). Further analysis of wound images revealed improved wound closure in all BD groups compared to the control group, with the 1.0 and 1.2 mm defects exhibiting more pronounced enhancement (Fig. S10c, d). Histological examination by H&E staining indicated incomplete wound closure on day 7 in both the control and 0.5 mm groups, with gaps remaining between newly formed epidermal edges. In contrast, wounds in the 1.0 mm and 1.2 mm defect groups exhibited complete re-epithelialization (Fig. S11a, b). Immunofluorescence staining for cytokeratin provided clear visualization of epidermal regeneration, consistent with observations from H&E staining (Fig. S11d). By day 14, wounds in all groups had fully healed, and no significant differences in scar distances were observed (Fig. S11a, c). In summary, an appropriately sized BD is essential for promoting diabetic wound healing. Smaller defects provide limited therapeutic benefit, whereas excessively large defects may introduce risks such as fractures.

Given the pivotal role of PDGF-BB in mediating vascular regeneration, we further investigated the regeneration of CD31^hi^ Emcn^hi^ vessels in diabetic foot skin wounds following BD intervention. Flow cytometry analysis showed that on day 7 post-wounding, there was a significant increase in the proportion of CD31^hi^ Emcn^hi^ cells in the BD group compared to the control group (Fig. 4g). Immunofluorescence staining also confirmed greater amounts of CD31^hi^ Emcn^hi^ endothelial cells in wound tissues (Fig. 4h). Next, to better understand the function of new blood vessels, we examined the proliferative activity of endothelial cells. The results showed that the BD induced more proliferated endothelial cells in wound beds versus control, and a high-density distribution of Edu^+^ proliferated cells was present around the vessels in the BD group (Fig. 4i). Furthermore, in vitro tube formation experiments conducted with endothelial cells revealed that peripheral blood plasma from the BD stimulated more tube formation of HUVECs than the control plasma (Fig. S12). Together, these results suggest that BD-induced increases in plasma PDGF-BB protein were involved in angiogenesis and accelerated skin wound healing.

### PDGF-BB promotes dermal fibroblasts-keratinocyte crosstalk

**Fig. 5:**
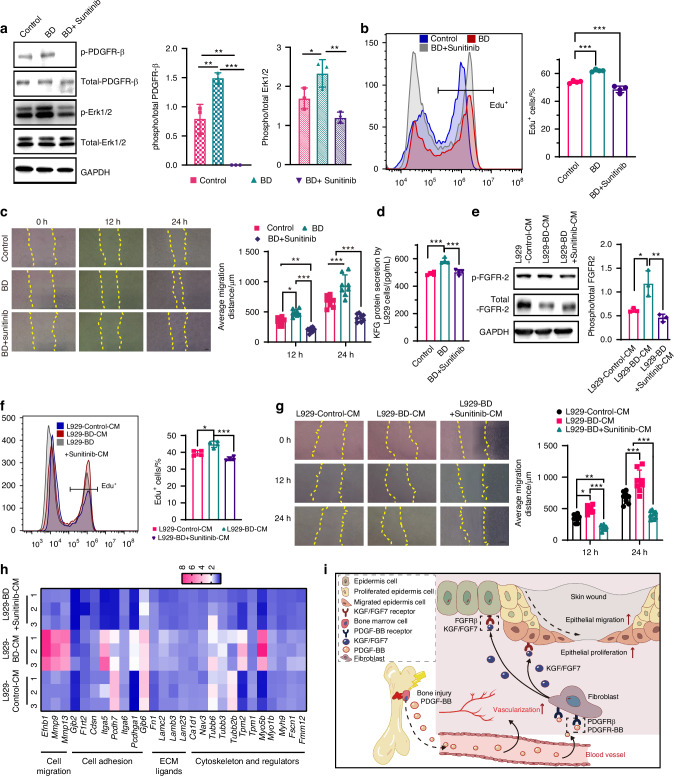
PDGF-BB enhances the crosstalk of fibroblasts and epidermal cells. **a** Western blot analysis and the relative level of phosphorylated FGFR-2 and Erk1/2 in L929 cells after treating with the combining 5% control plasma (Control), 5% bone defect plasma (Bone defect) and 5% bone defect plasma combining1uM Sunitinib (Bone defect + Sunitinib) for 20 min (*n* = 3). **b** Quantitative statistics of the percentage of Edu^+^ L929 cells treated with indicated plasma by flow cytometry for 48 h (*n* = 4). **c** The average migration distance of L929 cells in different groups was quantitatively analyzed according to scratch test (*n* = 8). Scale bar, 200 μm. **d** KGF protein expressions of L929 cell conditional medium by Elisa (*n* = 4). **e** Western blot analysis and the relative level of phosphorylated FGFR-2 and Erk1/2 in HaCaT after treating with the indicated L929 CM for 20 min (*n* = 3). **f** Quantitative statistics of the percentage of Edu^+^ HaCaT cells by flow cytometry. Cells were treated with L929-Control-CM, L929-BD-CM, and L929-BD+Sunitinib-CM, respectively for 48 h (*n* = 4). **g** The average migration distance of HaCaT cells treated with differenced CM was quantitatively analyzed according to scratch test. Yellow dashed line marks scratch wound edges (*n* = 8). Scale bar, 200 μm. **h** Heatmap showing the relative mRNA expression of HaCaT cells by qPCR (*n* = 3). The values were the multiple of the gene expression of each gene relative to that of 10% FBS treated HaCaT cells. **i** Illustration of the mechanism of skin wound healing induced by bone defect. Data are presented as mean ± SD and statistical significance was analyzed via one-way ANOVA with Tukey’s multiple comparison test for (**a**, **b**, **d**, **e**, **f**), two-way ANOVA with Tukey’s multiple comparison test for (**c**, **g**). *P* value: *P < 0.05, **P < 0.01, ***P < 0.001

During the wound healing process, PDGF-BB not only contributes to angiogenesis but also regulates the proliferation, migration, and collagen deposition of fibroblasts,^37^ and has a certain regulatory effect on their production of KGF.^29^ Although IL-1β is the most effective factor to promote the secretion of KGF from fibroblasts,^38^ we did not observe significant differences in IL-1β protein expression of plasma and skin wound tissues between the control and the BD groups (Fig. S13). We further explored whether PDGF-BB in plasma is crucial for the re-epithelialization of epidermal cells mediated by fibroblasts. To this end, we used an inhibitor (sunitinib) to antagonize PDGFRβ signaling and studied its impact on the crosstalk between fibroblasts and epidermal cells. Our analysis of the phosphorylation level of PDGFRβ in L929 cells revealed that BD plasma could activate PDGFRβ phosphorylation compared to control plasma. However, sunitinib significantly reduced PDGFRβ phosphorylation and also partially inhibited the phosphorylation of Erk1/2 (Fig. 5a). Correspondingly, the inhibition of PDGFRβ signaling eliminated the promoting effect of BD plasma on fibroblast proliferation and migration and had a certain inhibitory effect compared to control plasma (Fig. 5b, c). Furthermore, we found that inhibiting PDGFRβ signaling diminished the stimulatory effect of BD plasma on KGF protein secretion by L929 cells, showing no significant difference when compared to the control plasma (Fig. 5d).

Then, we collected L929 conditioned medium to culture HaCaT and found that L929-BD+Sunitinib-CM inhibited the phosphorylation of FGFR-2 compared to L929-BD-CM, which was similar to the level of L929-control-CM (Fig. 5e). Accordingly, the effect of L929-BD+Sunitinib-CM on HaCaT proliferation and migration was significantly weaker than that of L929-BD-CM, returning to the level of L929-control-CM (Fig. 5f, g). We also compared the expression of mRNA related to cell migration, cell adhesion, ECM ligands, and cytoskeleton and regulators. The mRNA expression profile of L929-control-CM was more similar to that of L929-BD+Sunitinib-CM. L929-BD-CM mainly increased the expression of *Efnb1*, *Mmp3*, and *Mmp13* mRNA related to HaCaT cell migration, *Itga5* mRNA related to cell adhesion, and *Tpm2*, *Myo1b* mRNA related to cytoskeleton and regulators,^39^ with no significant effect on the expression of mRNA related to ECM ligands (Fig. 5h). In summary, our results suggest that the elevated levels of PDGF-BB in plasma induced by BD activate its receptor signaling on fibroblast, which in turn mediates the paracrine secretion pathway of fibroblasts to secrete KGF accelerating re-epithelialization of diabetic wounds (Fig. 5i).

### Collagen hydrogels carrying bone derived factors efficiently promote diabetic wound healing

**Fig. 6:**
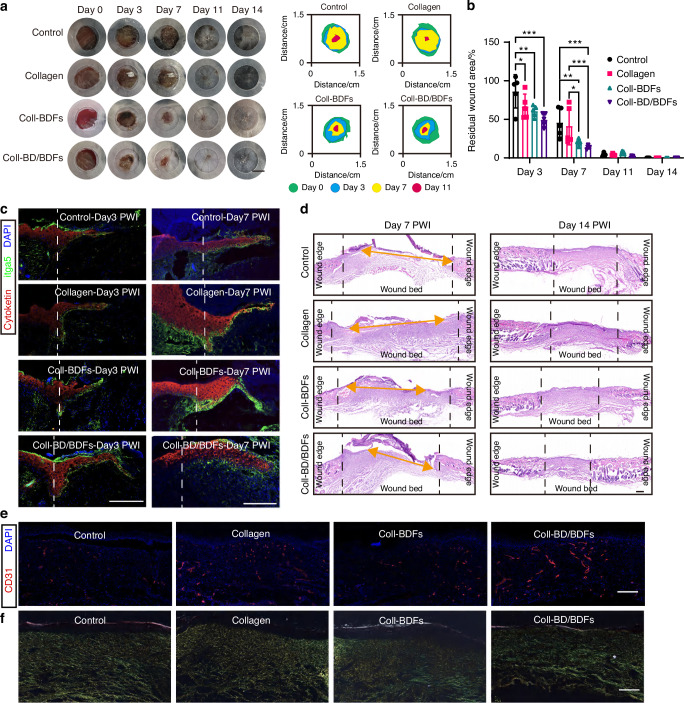
Bone derived factors from bone defect diabetic mice efficiently promote diabetic wound healing. **a** Representative images of dorsal wound treated with Tegaderm dressing (Control), Collagen hydrogel (Collagen), Collagen hydrogel containing bone-derived factors (Coll-BDFs), and Collagen hydrogel containing bone-derived factors of diabetic bone defect mice (Coll-BD/BDFs) at different times. Scale bar, 5 mm. **b** Quantification of the percent of dorsal wound residual area at indicated times in different groups (*n* = 6). **c** Sagittal sections of dorsal wound tissue immunolabeled for cytokeratin (red) and integrin a5 (green), cell nucleus for DAPI (blue) at days 3 and 7. Scale bar, 200 μm. **d** Representative H&E staining of dorsal wound tissue on days 7 and 14. Scale bar, 200 μm. **e** Sagittal sections of day 14 wounds tissue immunolabeled for CD31 (red) and cell nucleus for DAPI (blue). Scale bar, 200 μm. **f** Representative Sirius red staining of dorsal wound tissue on day 14. Scale bar, 200 μm. Data are presented as mean ± SD and statistical significance was analyzed via two-way ANOVA with Tukey’s multiple comparison test for (**b**). *P* value: *P < 0.05, **P < 0.01

In order to identify the therapeutic potential of bone-derived factors, such as PDGF-BB, released from damaged bone to promote skin wound healing, we collected bone marrow supernatant from both injured tibia and the contralateral uninjured tibia, both of which are rich in bone-derived factors. We created an 8 mm full-thickness skin wound on the back of diabetic mice as a skin injury model. The models were treated with collagen hydrogel (collagen group), collagen hydrogel loaded with the contralateral uninjured bone marrow supernatant (Coll-BDFs), and collagen hydrogel loaded with BD bone marrow supernatant (Coll-BD/BDFs). The control group wounds were treated with Tegaderm^TM^ dressing. Through photographic analysis of wound closure, we found that both hydrogels containing bone-derived factors could accelerate the closure of diabetic wounds, with a significant decrease in the residual wound area on days 3 and 7 compared to the control and collagen hydrogel groups (Fig. 6a, b).

There was no significant difference in wound closure between the Coll-BDFs and Coll-BD/BDFs groups, prompting us to further analyze histological differences in the wounds. Immunofluorescence analysis of epidermal cell migration in wound bed on days 3 and 7 revealed that wounds treated with Coll-BD/BDFs had significantly longer cytokeratin^+^ integrin a5^+^ migrating tongues than other groups (Fig. 6c). H&E images of wound tissue also showed that on day 7 post-injury, the epidermal gap was smallest in the Coll-BD/BDFs group, followed by the Coll-BDFs group, while it was relatively larger in the control and collagen hydrogel groups. The Coll-BD/BDFs significantly reduced scar tissue area compared to other groups on day 14. Although all wounds had completed re-epithelialization on day 14, the thickness of newly formed epidermis in the Coll-BD/BDFs group was smaller and closer to normal epidermal thickness (Fig. 6d). Immunofluorescence staining of CD31 in wound tissue also showed that Coll-BD/BDFs could induce more blood vessel regeneration than other groups, and the wound vascular regeneration was also improved following treatment with Coll-BDFs (Fig. 6e).

During the wound healing and remodeling phase, the transition from type I to type III collagen is crucial for preventing scar formation. Therefore, we used Sirius Red staining to analyze collagen distribution in wound tissue on day 14. The results showed that large amounts of type I collagen fibers (yellow) were still distributed in the wound tissue of the control and collagen groups, with less type III collagen (green). In addition to observable type I collagen, there was a significant increase in type III collagen in the wound tissue of the Coll-BDFs group. In the wounds of the Coll-BD/BDFs group, type III collagen was dominant, with less type I collagen, suggesting potential mitigation of scar formation (Fig. 6f). Taken together, these findings confirmed that hydrogels containing bone-derived factors have potent potential to improve diabetic skin wound healing, and that bone-derived factors produced by injured bone tissue can enhance the healing effect on diabetic wounds.

## Discussion

Severe DFUs, due to their large wound area and depth, limit the effectiveness of local in-situ wound treatment. Promoting wound healing by mobilizing the body’s endogenous healing mechanisms is a promising therapeutic method. Although clinical results have shown that changes in the bone microenvironment can accelerate wound healing in diabetic patients’ feet, the specific mechanism of how bone interacts with distal skin remains unclear.^15–17^ In this study, we proposed that a significant amount of PDGF-BB is produced in the bone marrow after a tibial defect, then circulated to the foot wound site via peripheral blood, participating in diabetic wound healing. PDGF-BB activates the paracrine pathway of fibroblasts through PDGFRβ signal, leading to enhanced proliferation and migration of epidermal cells. Specifically, we found the diabetic foot wound treated with BD had been completely re-epithelialized on day 7. Additionally, PDGF-BB in plasma can also promote the regeneration of CD31^hi^ Emcn^hi^ blood vessels in the skin wound. Moreover, we found that hydrogel loaded with bone-derived factors such as enriched PDGF-BB, indeed accelerate diabetic dorsal wound healing, demonstrating the potential application of bone-derived factors in wound repair. Taken together, our findings have established a clear link between injured bone and wound healing via the secretion of bone-derived factors.

There exists extensive and frequent cross-organ communication between different organs. Cross-organ communication signals such as the liver-bone axis, the nerve-skin axis, and the nerve-vascular axis have been proven to be crucial for maintaining homeostasis and promoting tissue regeneration.^40–44^ It is an exciting research field to improve diabetic wound treatment effectiveness through effective intervention of cross-organ communication signals between bone and skin. Bone and wound tissues communicate through multiple pathways, including neurological intervention and immunological modulation, with blood-mediated interaction representing perhaps the most direct form of communication. Bone tissue secretes growth factors and releases exosomes that can influence skin tissue regeneration via peripheral circulation. Recent research has demonstrated that miR-494-3p and antioxidants in exosomes released during bone transport significantly contribute to wound healing.^45^ In our study, we have identified bone-derived growth factors as another crucial mediator in activating tissue regeneration at wound sites.

Through proteomics analysis, we observed significant alterations in protein factors present in peripheral blood following BD, with PDGF-BB expression exhibiting a marked increase. Chronic diabetic wounds exhibit delayed healing due to a deficit and diminished vitality of growth factors. In contrast to conventional approaches that involve the exogenous supplementation of growth factors to compensate for their insufficient levels in wounds,^46,47^ we employ tibia defect to stimulate bone regeneration, thereby augmenting the secretion of endogenous factors. The elevated levels of PDGF-BB in plasma following BD can persist for ~7 days, ensuring sustained growth factor delivery via blood circulation and addressing the inadequate secretion of bioactive factors in diabetic wounds. Notably, various cell types, including macrophages and preosteoclasts, are capable of producing PDGF-BB during bone regeneration. In vitro cellular studies have demonstrated that preosteoclasts exhibit a significantly higher secretion rate of PDGF-BB than monocytes/macrophages.^36^ Consequently, we hypothesize that the increase in PDGF-BB protein levels in plasma and wound tissue may be attributed to the activation of preosteoclasts at the site of BD.

PDGF-BB represents a pivotal factor in promoting vascular regeneration,^48^ and the elevated PDGF-BB levels in both plasma and wound tissue, triggered by BD, emerge as a crucial determinant of enhanced wound vascularization. Furthermore, our study reveals that PDGF-BB promotes rapid wound re-epithelialization through indirect mechanisms rather than direct action on epidermal cells. Considering that skin wound healing is a precise process involving multiple cells interacting with each other, multiple kinds of cells influence the behavior of epidermal cells, such as macrophages, fibroblasts, and other cells secrete EGF, IL-6, and KGF to promote the migration and proliferation of epidermal cells.^49–51^ In vitro cellular inhibition experiments confirmed that high expression of PDGF-BB in plasma participates in fibroblast and epidermal cell crosstalk by increasing fibroblast secretion of KGF.

Restoration of tissue function and coordination of multi-system repair are critical to the host and represent an extraordinary medical challenge. Exploring cross-organ interactions has the potential to provide new ideas for the treatment of diabetic wound repair. The mechanisms by which bone facilitates multi-tissue regeneration are complex and remain poorly understood. Our study identified a mode of endocrine regulation of skin wound healing by bone, mediated by the bone-derived secretory factor PDGF-BB. While mice and humans indeed differ in skin structure, gene expression patterns, and healing capacities, it is noteworthy that PDGF-BB is a highly conserved gene with considerable consistency in expression patterns between mice and humans.^52^ This conservation provides a reasonable foundation for using mouse models to study the role of bone-derived PDGF-BB in wound healing processes. These results also inspire us to actively stimulate bone secretion of PDGF-BB to achieve similar effects. For example, moderate mechanical force stimulation^53^ and low intensity pulsed ultrasound^54^ can promote PDGF-BB expression, which may enhance wound healing. Given their role in promoting angiogenesis and tissue regeneration, bone-derived factors may represent novel targets for the treatment of chronic wounds and ischemic diseases.

## Materials and methods

### Animal models

Male C57BL/6J mice, aged 7–8 weeks, were obtained from JSJ-Lab Co. Ltd (Shanghai, China). Ethical approval for this study was obtained from the Ethics Committee of the East China University of Science and Technology (Approval No. ECUST-2023-039). In order to establish an animal model of diabetes, C57BL/6J mice were administered intraperitoneal injections of STZ (Macklin, China) dissolved in sodium citrate buffer (pH = 4.5) daily for 5 days, at a dosage of 50 mg per kg of body weight. Following an additional 2 weeks, mice with blood glucose levels exceeding 20 mmol/L were identified as diabetic mice for subsequent experiments.

To evaluate glucose tolerance in STZ-induced mice, we performed intraperitoneal glucose tolerance tests (IPGTT). Non-diabetic wild-type (WT) mice served as controls. Briefly, mice were fasted for 12 h, followed by measurements of body weight and baseline blood glucose levels. A 20% glucose solution (D-(+)-Glucose dissolved in distilled water) was administered intraperitoneally at a dose of 1 g glucose per kg body weight. Blood glucose levels were monitored at 15, 30, 60, 90, and 120 min post-injection, and glucose curves were generated and analyzed.

For BD model, the skin and muscles overlaying the mouse tibia were meticulously peeled off to expose the tibia. A hole with a diameter of 1 mm, or other sizes as required by the experimental design, was then precisely drilled into the anteromedial surface of the tibia using a high-speed drill. The drilling site was flushed with saline to remove bone debris. Subsequently, the surrounding muscles and skin were sutured. The operation for the sham group was the same as that for the BD group, except for bone drilling.

For foot full-thickness wounding, after the standard anesthesia procedure, right side foot skin was swabbed with ethanol prior to wounding. 3 mm biopsy punches were used to make full-thickness wounds on the dorsal foot of mice. The process of wound closure was documented through photographs on days 3, 7, 11, and 14 post-injury. These images were further processed using Image J software for wound area measurements.

For Full-thickness wounding of the dorsal skin, dorsal hairs were shaved with clippers and skin was swabbed with ethanol prior to wounding. Two round wounds with a diameter of 8 mm were made on the back of each mouse. The wound was treated with Tegaderm^TM^ dressing (Control group), collagen hydrogel (Collagen group), collagen hydrogel loaded with contralateral uninjured bone marrow supernatant from diabetic mice (Coll-BDFS group), and collagen hydrogel loaded with BD bone marrow supernatant (Coll-BD/BDFs group), respectively. On days 3, 7, 11, and 14, the wounds were photographed and wound areas were measured by Image J software.

### Histological evaluation

At days 7 and 14 post-wounding, the mice were euthanized, and the wound tissue was carefully excised. The tissue was then fixed in formaldehyde for 4 h, followed by a dehydration process in an ascending series of ethanol concentrations, and finally embedded in paraffin wax. Histological sections of 4.5 μm thickness were prepared using a microtome. For histological examination, the sections were stained with H&E and Sirius Red staining, following standard protocols. Photomicrographs were obtained using an Olympus VS200 virtual slide microscope. The subsequent quantitative analysis was conducted utilizing ImageJ software.

### Immunofluoresence staining

The skin wound tissue was collected at various time intervals and fixed in 4% paraformaldehyde at 4 °C for 4 h. Post fixation, the samples were immersed in a solution comprising 20% sucrose and 2% polyvinylpyrrolidone overnight, after which they were embedded in OCT compound (Leica) and sectioned into slices of 10 μm thickness. The sections underwent permeabilization with 0.1% Triton X-100 in PBST for 20 min at room temperature, followed by blocking with 5% donkey serum for 1 h at room temperature. For immunofluorescence, primary antibodies recognizing Emcn (Santa Cruz, V.7C7, 1:50), CGRP (Invitrogen, PA185250, 1:100), Cytokeratin (Abcam, ab9377, 1:100), CD31 (Abcam, ab182981, 1:100), and integrin α5 (BD Pharmingen, 553319, 1:100) were deployed. Visualization of primary antibodies was accomplished with appropriate secondary antibodies: Donkey anti-rabbit Alexa Fluor 647, Donkey anti-Rat Alexa Fluor 594, and Donkey anti-goat Alexa Fluor 594.

To label mitotic cells with EdU, an intraperitoneal injection of EdU was administered at a dosage of 200 mg/kg prior to the collection of tissue samples. The EdU click-it reaction was performed as the manufacturer’s instructions (Beyotime), post the secondary antibody incubation. The sections were mounted with ProLong gold anti-fade reagent with DAPI (Cell Signaling Technology) before imaging, and a minimum of five different microscopic images were examined using a Leica Sp8 confocal laser scanning microscope.

### Flow cytometry

The wound beds and skin surrounding the wound edges were excised and digested into single cells using a blend of collagenase I, neutral protease, and DNases, following established protocols. For macrophages and endothelial cells analysis, cells were resuspended in staining buffer and incubated with a series of antibodies for 30 min at 4 °C. CD45 (Biolegend, FITC; 1:200), F4/80 (Biolegend, PE/Cy7; 1:200), CD86 (Biolegend, PE; 1:200), CD206 (Biolegend, BV421; 1:200), CD11b (Biolegend, AF700; 1:200), CD31 (Biolegend, BV650; 1:100), and Ter119 (BD Biosciences, PE/Cy5; 1:100). Dead cells were excluded using a live/dead fixable far red dead cell stain kit (Thermo Fisher Scientific, USA) as per the manufacturer’s protocol prior to analysis using a Cytoflex LX (Beckman Coulter). The flow cytometry results were analyzed using FlowJo software. The specific gating strategies for macrophage and angiogenesis analysis can be found in Figs. S14 and S15.

### Plasma samples

24 h post-BD surgery, all diabetic mice were anesthetized and peripheral blood was drawn into a tube containing EDTA as an anticoagulant. Control plasma was obtained from diabetic mice that did not undergo BD surgery. The samples were immediately centrifuged at 4 °C and 2 000 r/min for 10 min. The supernatant was meticulously collected, transferred to a fresh tube, and stored at −80 °C for subsequent use.

### Cell proliferation analysis

L929 fibroblasts and HaCaT cells (ATCC, USA) were cultured in DMEM supplemented with 10% fetal bovine serum (FBS) at 37 °C in a 5% CO_2_ atmosphere. For cell proliferation analysis, 1 × 10^5^ L929 cells or HaCaT cells were seeded in each well of a 6-well plate and incubated for 12 h. The culture medium was then replaced with 10% FBS containing 5% plasma or conditioned medium, and the cells were incubated for 48 h. The blank group consisted of cells incubated with DMEM containing 10% FBS. EdU was added to the culture medium 2 h before harvest. The cells were digested into single cells using trypsin, collected into a centrifuge tube, then fixed and permeabilized for 20 min. The EdU click-it reaction was performed according to the manufacturer’s instructions (Beyotime), and the proportion of EdU^+^ cells was measured using a Cytoflex LX flow cytometer.

### Cell migration analysis

To evaluate the migratory capacity of L929 cells and HaCaT cells, a scratch wound healing assay was performed in 24-well plates. A total of 1 × 10^5^ cells per well were seeded and cultured for 24 h, then a vertical scratch wound was created using a sterile pipette tip. The wells were then gently washed twice with PBS to remove cell debris, and the cells were cultured in 2% FBS combined with plasma or conditioned medium. DMEM with 2% FBS was used as a blank control. At 0 h, 12 h, and 24 h after injury, the cells were photographed under an inverted microscope (Leica), and the distance that the migrated cells had infiltrated was quantitatively evaluated using ImageJ software.

### qPCR

L929 cells or HaCaT cells were cultured with different culture medium in a 6-well plate. After 3 days, total mRNA was extracted from the cells using Trizol reagent (Takara Biotechnology, Dalian, China) and reverse-transcribed into cDNA using the Prime-Script RT reagent kit (Takara Biotechnology, Dalian, China) following the manufacturer’s instructions. Quantitative PCR (qPCR) was performed using TB-Green on the CFX96 TouchTM PCR detection system (Bio-Rad, USA). The primer sequences for specific genes can be found in Tables S1 and S2. The results were normalized to the mRNA levels of *Gapdh*, as previously described, and all samples were assayed in triplicate.

### Preparation of conditioned media from L929 fibroblasts

L929 cells were stimulated in DMEM containing 10% FBS and control plasma or BD plasma for 3 days. The blank group consisted of cells incubated with DMEM containing 10% FBS. After stimulation, the cell plates were washed with PBS, and conditioned media were collected after an additional day of culture with FBS-containing medium. The conditioned media were then centrifuged at 300 × *g* for 10 min at 4 °C and stored at −80 °C until further use. For cell proliferation experiments, 10% FBS conditioned media were used. For migration experiments and Western blot assays to detect growth factors, 2% FBS conditioned media were used. In some experiments, PDGFRβ signaling inhibitor (sunitinib, 1 μg/mL) were added to the BD plasma to stimulated L929 cells and collected conditioned media (L929-BD+Sunitinib-CM).

### Elisa

The levels of KGF, EGF, or IL-6 proteins in the conditioned medium were measured using a Mouse KGF Quantikine ELISA kit (ElAab), a Mouse EGF or a Mouse IL-6 Quantikine ELISA kit (Neobioscience). The levels of KGF or PDGF-BB protein expression in wound tissue or bone marrow supernatant were measured using a Mouse KGF Quantikine ELISA kit (ElAab) or a Mouse PDGF-BB Quantikine ELISA kit (Neobioscience). All ELISA kits were conducted according to the manufacturer’s instructions.

### Western blot analysis

To investigate the effects of plasma or conditioned media on L929 cells or HaCaT cells, cells were starved overnight in 2% FBS before being stimulated with 5% plasma or conditioned media for 20 min. The cells were then lysed in 1 × RIPA buffer containing phenylmethylsulfonyl fluoride (PMSF) for 20 min on ice. Total protein was quantified using a Pierce BCA protein quantification kit, and 30 μg of total protein lysates were loaded onto a 4%–20% SDS-polyacrylamide gel electrophoresis (PAGE). The separated proteins were transferred to a polyvinylidene fluoride membrane (Millipore), blocked for 30 min with 5% milk in TBS-T, and incubated with primary antibodies recognize PDGFR-β (#3169), p-PDGFRβ (#3124), Erk1/2 (#4695,), p-Erk1/2 (#4370), p38 (#8690), p-P38 (#4511), FGFR-2 (#23328), p-FGFR-2 (#3476), and GAPDH (#5174) at 4 °C overnight. All primary antibodies were used at a dilution of 1:1 000 and purchased from Cell Signaling. The membranes were then washed in TBS-T and incubated with HRP-coupled secondary antibodies at room temperature. The proteins were detected using a chemiluminescence imaging system (Tanon, Shanghai, China), and the total intensity of each band was quantified using ImageJ software.

### Proteomics

We collected plasma from diabetic mice 1 day after BD and plasma from diabetic mice without BD (Control). The samples were immediately stored in liquid nitrogen. Protein analysis was conducted by Shanghai Applied Protein Technology Co. Ltd (Shanghai, China). Initially, equal amounts of the samples were pooled together to create a pool sample for HPRP fractionation. The pool sample was then subjected to LC-MS/MS (timsTOF_DDA mode) analysis to generate a DDA library database. Subsequently, each individual sample was analyzed separately using LC-MS/MS (timsTOF_DIA mode), and the aforementioned DDA database was utilized for qualitative and quantitative analysis. For the DDA library data, we employed Spectronaut^TM^ 14.4.200727.47784 (Biognosys) software to search the FASTA sequence database, which was downloaded from the website: http://www.uniprot.org.

To identify homologous sequences, we locally searched the studied protein sequences using the NCBI BLAST^+^ client software (ncbi-blast-2.2.28 + −win32.exe) and InterProScan. GO terms were then mapped, and sequences were annotated using Blast2GO software. The GO annotation results were visualized using R scripts. Following annotation, we conducted a blast search of the studied proteins against the KEGG database (http://geneontology.org/) to retrieve their KEGG classification and pathway mappings.

To identify significant functional categories and pathways, we conducted an enrichment analysis using Fisher’s exact test, with the whole quantified protein dataset as the background. We further applied the Benjamini-Hochberg correction to adjust the derived *P*-values for multiple testing. Only functional categories and pathways with *P*-values below a threshold of 0.05 were deemed significant. In addition, we identified differentially expressed proteins based on a fold change of ≥1.5-fold and a *P* value < 0.05.

### Intravenous injection of PDGF-BB

On days 1 and 3 following the induction of foot wounds in diabetic mice, the animals were anesthetized, and PDGF-BB solution was administered via retro-orbital intravenous injection. The injection volume was calculated based on a total blood volume equivalent to 7% of the mouse’s body weight, with a low concentration of PDGF-BB set at 1 ng/mL and a high concentration at 10 ng/mL.

### HUVECs tube formation analysis

To prepare the culture plates, Matrigel (Corning) was precoated onto 96-well plates and allowed to gel by incubating at 37 °C for 30 min. HUVECs were seeded on the polymerized Matrigel at a density of 4 × 10^4^ cells per well using DMEM with 2% FBS and plasma or DMEM with 2% FBS as a negative control. After incubation for 4 h, cell sprouting was observed and recorded using an inverted microscope.

### Hydrogel scaffold preparation

The Collagen type I solution, which was purchased from Coring company, was dissolved in 0.02 mol/L acetic acid to obtain a concentration of 3.34 mg/mL. The pH of the solution was adjusted to 7.0 using 1.0 mol/L NaOH solution and vortexed for 2 min. The solutions were then poured into 24-well cell culture plates at a volume of 0.6 ml per well, frozen at −20 °C for 12 h, and freeze-dried for 24 h. To crosslink the freeze-dried scaffolds, they were immersed in a solution containing 33 mmol/L EDC and 6 mmol/L NHS in 75% ethanol for 24 h at 4 °C. After washing the scaffolds with ultrapure water five times, they were re-lyophilized. Finally, 50 μL of bone marrow supernatant was added to the hydrogel, freeze-dried, and stored at −20 °C.

### Statistical analysis

The statistical analysis of the results was performed using GraphPad Prism software (version 9.0; GraphPad, La Jolla, CA, USA). The data were presented as the mean ± standard deviation (SD). The number of biological replicates was indicated by *N*, and experiments were conducted at least three times unless otherwise specified. Unpaired, two-tailed Student’s *t*-tests were used for comparisons between two groups. For comparison of multiple experimental groups, either one-way analysis of variance (ANOVA) or two-way ANOVA was performed as appropriate. A P value less than 0.05 was considered statistically significant, indicated by asterisks as follows: *P < 0.05, **P < 0.01, ***P < 0.005, and ****P < 0.001.

## Supplementary information

Supplementary Materials

## Data Availability

All data associated with this study are present in the paper.
