# Supplementary material for: Injured bone-triggered osteokines secretion promotes diabetic wound healing
Source: Bone Res. 2025 Oct 2;13:83. doi: 10.1038/s41413-025-00454-9 (PMC12491472; doi:10.1038/s41413-025-00454-9)
Supplement: Supplementary file 1 — Supplementary Materials [file 41413_2025_454_MOESM1_ESM.docx]

Supplementary Materials for

**Injured bone-****triggered osteokines secretion promotes diabetic wound healing**

Tong Shen^1,2,^ ^†^, Kai Dai^2,3,4,^ ^†^, Shuang Zhang^1,2^, Jing Wang ^1,2,4, *^ & Changsheng Liu^2,3,4, *^

^1^State Key Laboratory of Bioreactor Engineering, East China University of Science and Technology, Shanghai, China.

^2^Engineering Research Center for Biomedical Materials of the Ministry of Education, East China University of Science and Technology, Shanghai, China.

^3^Key Laboratory for Ultrafine Materials of the Ministry of Education, East China University of Science and Technology, Shanghai, China.

^4^Frontiers Science Center for Materiobiology and Dynamic Chemistry, East China University of Science and Technology, Shanghai, China.

^†^These authors contributed equally: Tong Shen, Kai Dai

*Corresponding author: Jing Wang, Changshen Liu.

**Email:**  wangjing08@ecust.edu.cn ; [liucs@ecust.edu.cn](mailto:liucs@ecust.edu.cn)

**This file includes:**

Supplementary Text

Figs. S1 to S15

Tables S1 to S2


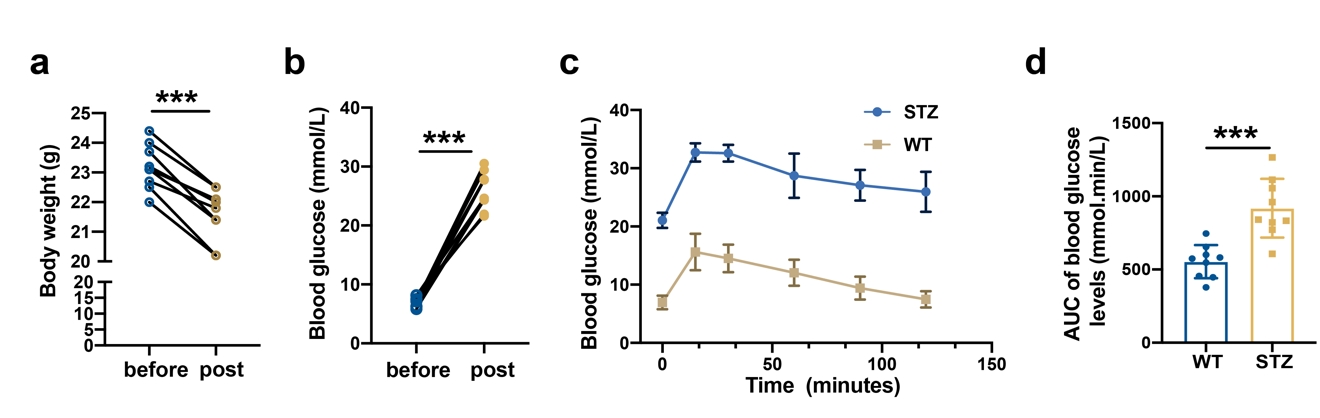


**Fig.S1. Assessment of diabetes-related parameters in STZ-induced mice. (**a) Body weight before and after STZ induction. (b) Blood glucose levels before and after STZ induction. (c) Blood glucose levels during glucose tolerance test in non-diabetic mice and STZ-induced mice. (d) Glucose area under the curve (AUC) during glucose tolerance test. Data are presented as mean ± SD and statistical significance was analyzed via unpaired two-tailed Student's t test. P value: *P < 0.05, **P < 0.01, ***P < 0.001.


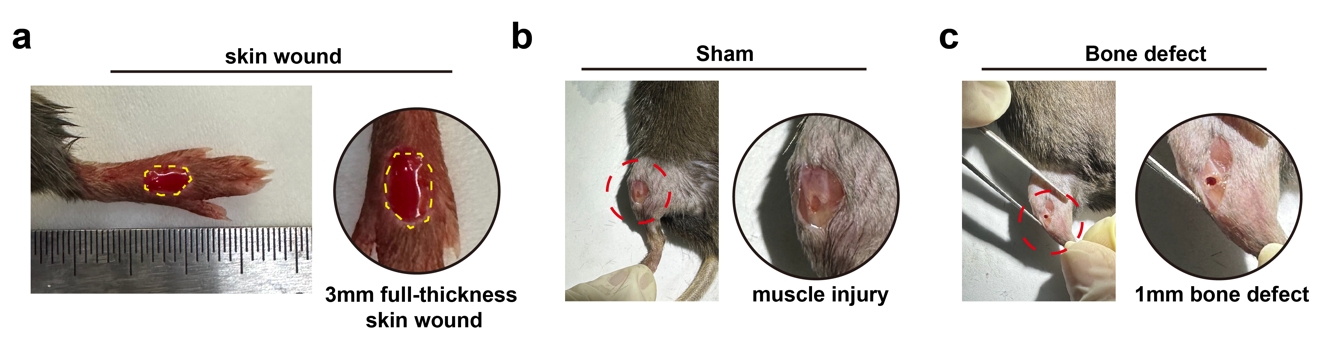


**Fig. S2. Surgical procedure photographs.** (a) Foot skin wound image. (b) Leg surgery image of the sham group. (c) Bone defect surgery image.

**
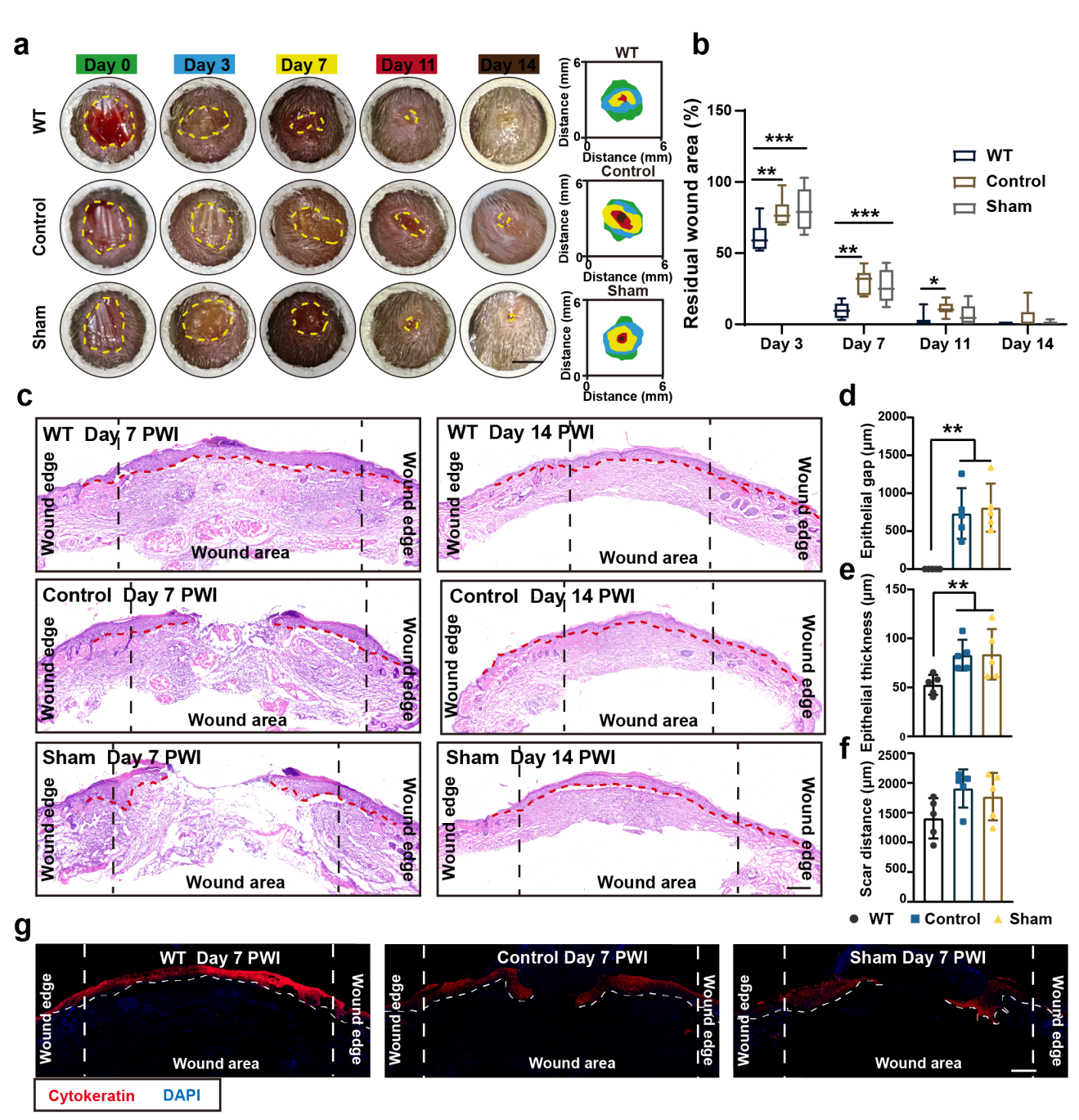
**

**Fig. S3. Wound healing effect evaluation.** (a) Representative images of foot wound healing in WT group(healthy), control group and sham group. Yellow dashed line represents remaining wound contour. Scale bar, 2 mm (b) Quantification of the percent of foot wound residual area at different times. (n = 8). (c) Representative H&E staining of foot wound tissue on day 7 and day 14. Scale bar, 200 μm. (d) Quantification of epidermis gap on day 7, (e) epidermal thickness on day 14, and (f) scar distance on day 14 according to HE staining images (n = 5). (g) Sagittal sections of day 7 wound tissue immunolabeled for cytokeratin (red) and DAPI (blue). Scale bar, 200 μm. Data are presented as mean ± SD and statistical significance was analyzed via two-way ANOVA with Tukey’s multiple comparison test for (b), one-way ANOVA with Tukey’s multiple comparison test (d), (e) and (f). P value: *P < 0.05, **P < 0.01, ***P <0.001.

**
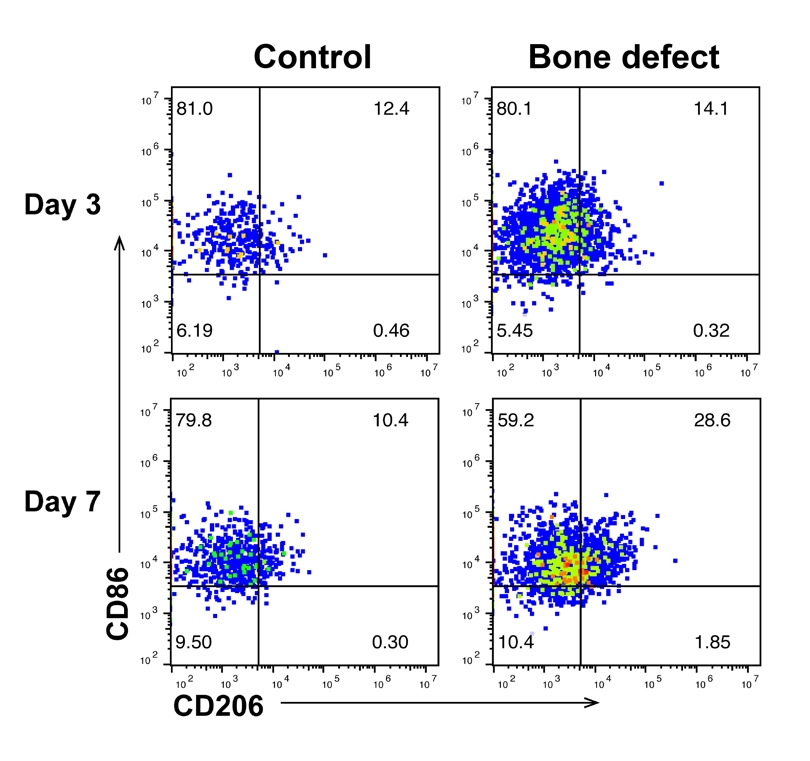
**

Fig. S4. Representative flow cytometry plots of macrophage subpopulations in diabetic wound tissue.


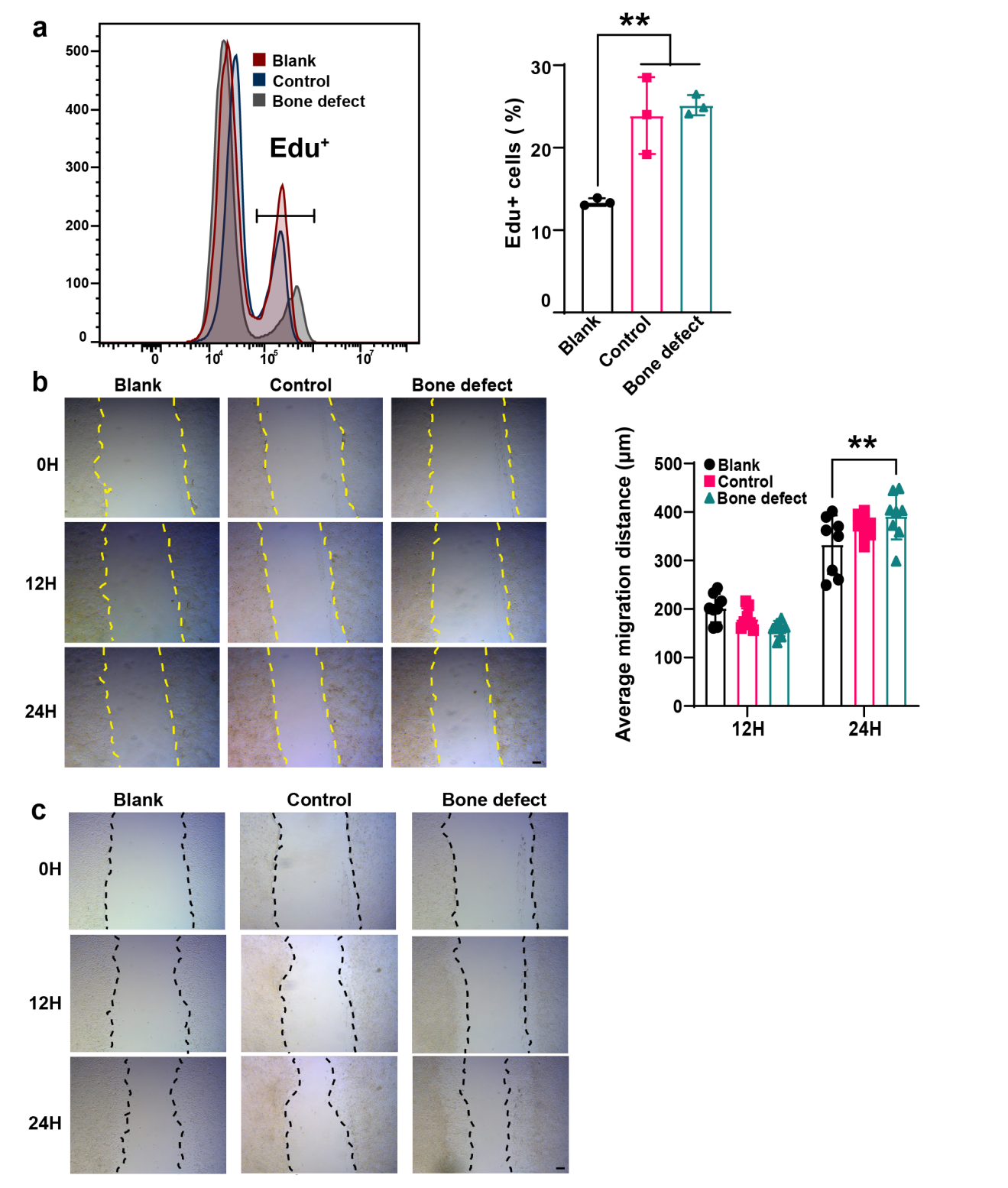


**Fig. S5.** **Plasma from bone defect diabetic mice did not affect the proliferation and migration of HaCaT cells.** (a) Quantitative statistics of the percentage of Edu^+^ HaCaT epidermal by flow cytometry. Cells were treated with 10% FBS (Blank) and 10% FBS combining 5% control diabetic mice plasma (Control) or bone defect diabetic mice plasma (Bone defect), respectively (n = 3) for 48h. (b) The average migration distance of HaCaT epidermal in different groups was quantitatively analyzed according to scratch test (n = 8). Yellow dashed line marks scratch wound edges. (c) The scratch typical images of L929 cells. Data are presented as mean ± SD and statistical significance was analyzed via one-way ANOVA with Tukey’s multiple comparison test for (a), two-way ANOVA with Tukey’s multiple comparison test for (b). P value: **P < 0.01.


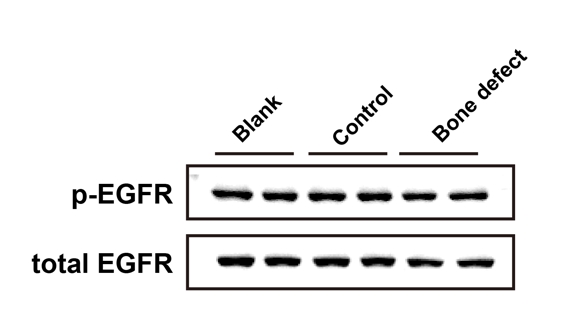


**Fig. S6. Western blot analysis and the relative level of phosphorylated EGFR in HaCaT cells after treating with the indicated CM for 20 min (n = 2)**


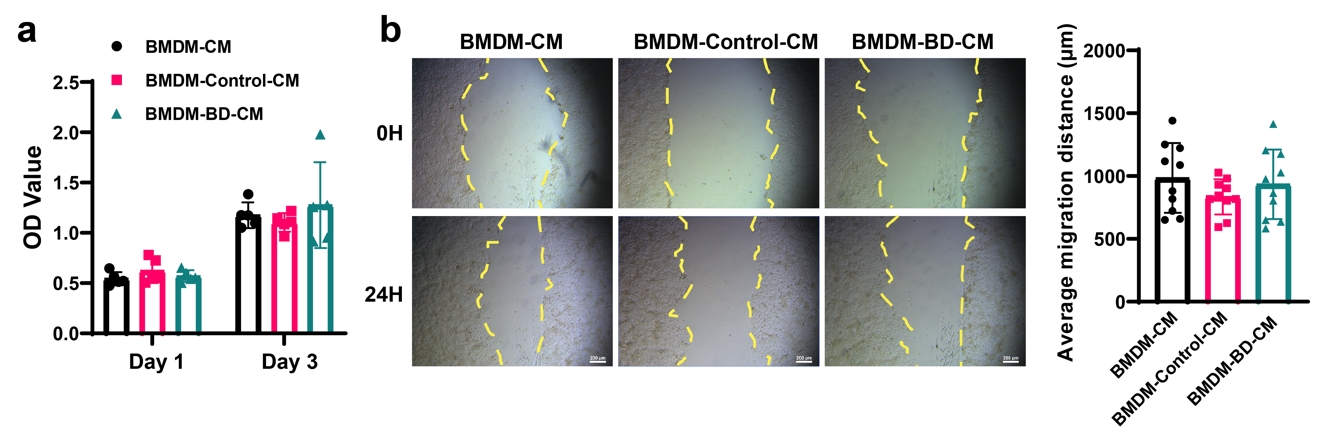


**Fig. S7. Macrophage conditioning after plasma stimulation had no significant effect on the proliferation and migration of HaCaT cells.** (a) Cell viability of HaCaT cells treated with differenced BMDM-CM at day 1and day 3. (b) The average migration distance of HaCaT cells treated with differenced BMDM-CM was quantitatively analyzed according to scratch test. Yellow dashed line marks scratch wound edges (n = 10). Data are presented as mean ± SD and statistical significance was analyzed via one-way ANOVA with Tukey’s multiple comparison test for (b), two-way ANOVA with Tukey’s multiple comparison test for (a).


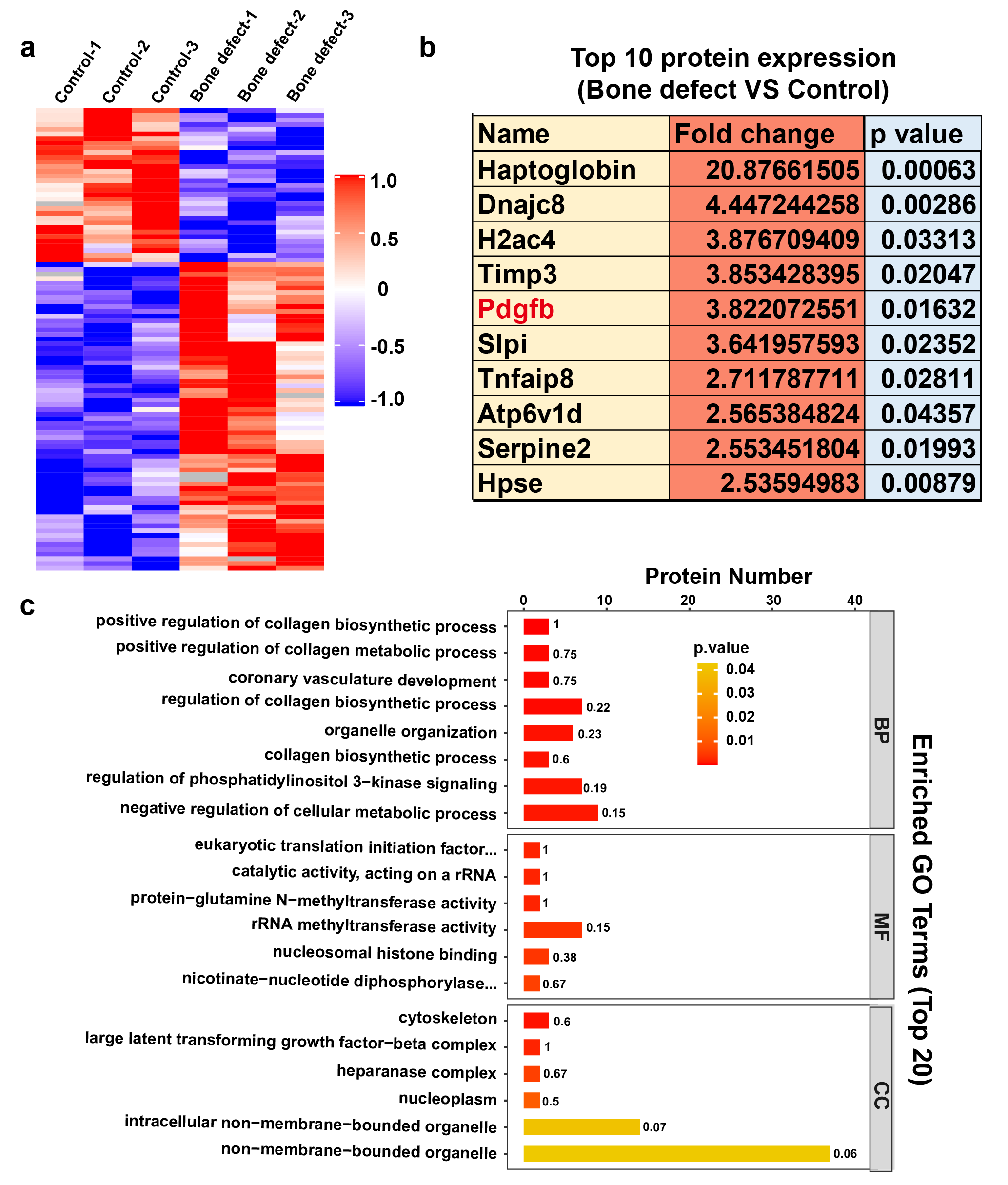


**Fig. S8. Proteomic analysis of diabetic plasma.** (a) Heatmap analysis of differentially expressed proteins. (b) GO analysis of differentially expressed proteins. (c) Top 10 upregulation differentially expressed proteins in bone defect plasma vs control plasma.


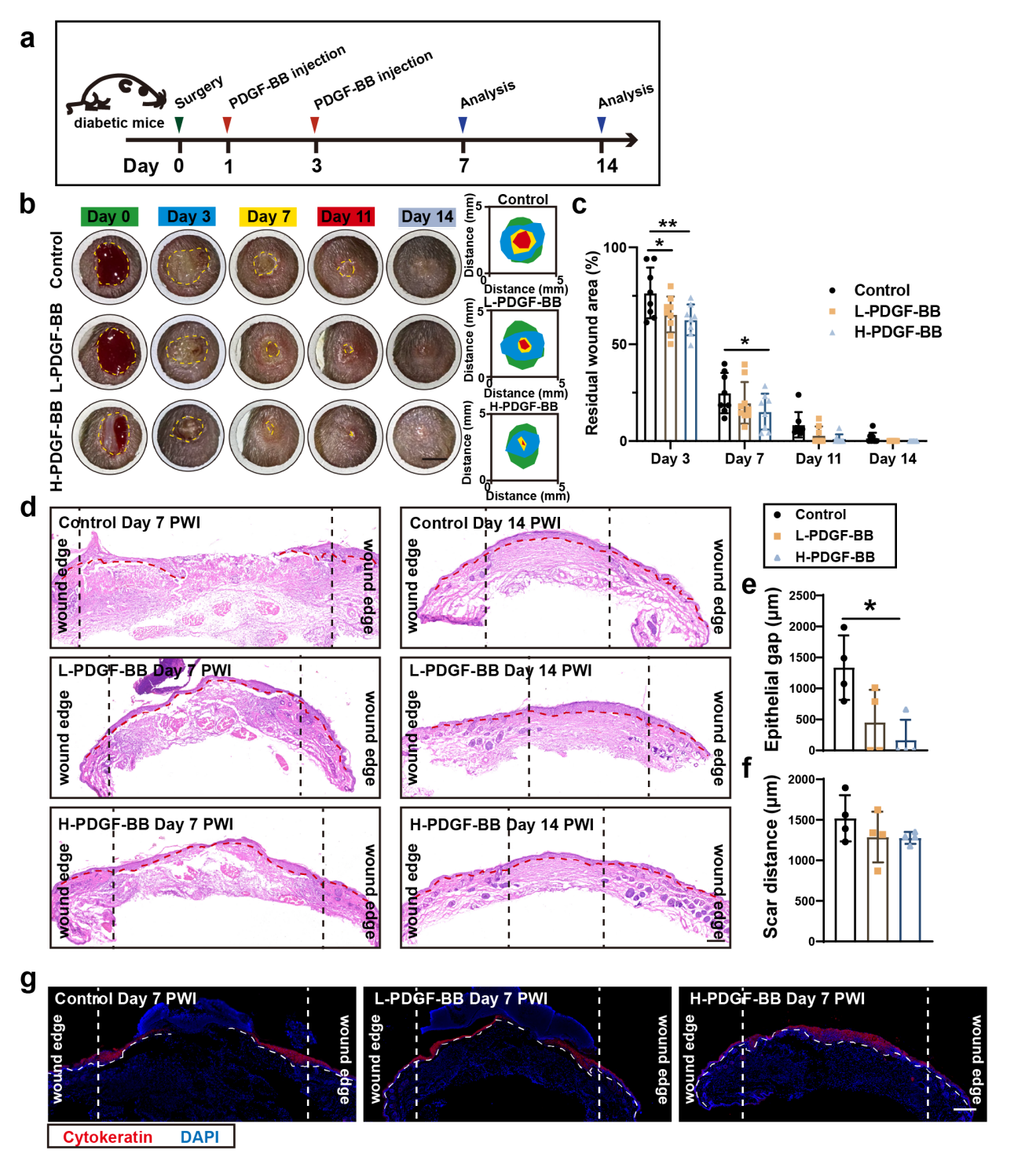


**Fig. S9. Evaluation of the effects of systemic delivery of PDGF-BB on diabetic foot wound healing.** (a) Schematic illustration of the surgical procedure involving retro-orbital venous injection of PDGF-BB. (b) Representative images of foot wound healing in control group, L-PDGF-BB group (1ng/ml PDGF-BB) and H-PDGF-BB group (10ng/ml PDGF-BB). Yellow dashed line represents remaining wound contour. Scale bar, 2 mm. (c) Quantification of the percent of foot wound residual area at different times. (n = 8). (d) Representative H&E staining of foot wound tissue on day 7 and day 14. Scale bar, 200 μm. (e-f) Quantification of epidermis gap (e) on day 7, scar distance (f) on day 14 according to H&E staining images (n=4). (g) Sagittal sections of day 7 wound tissue immunolabeled for cytokeratin (red) and DAPI (blue). Scale bar, 200 μm. Data are presented as mean ± SD and statistical significance was analyzed via two-way ANOVA with Tukey’s multiple comparison test for (c), one-way ANOVA with Tukey’s multiple comparison test for (e) and (f). P value: *P < 0.05, **P < 0.01.


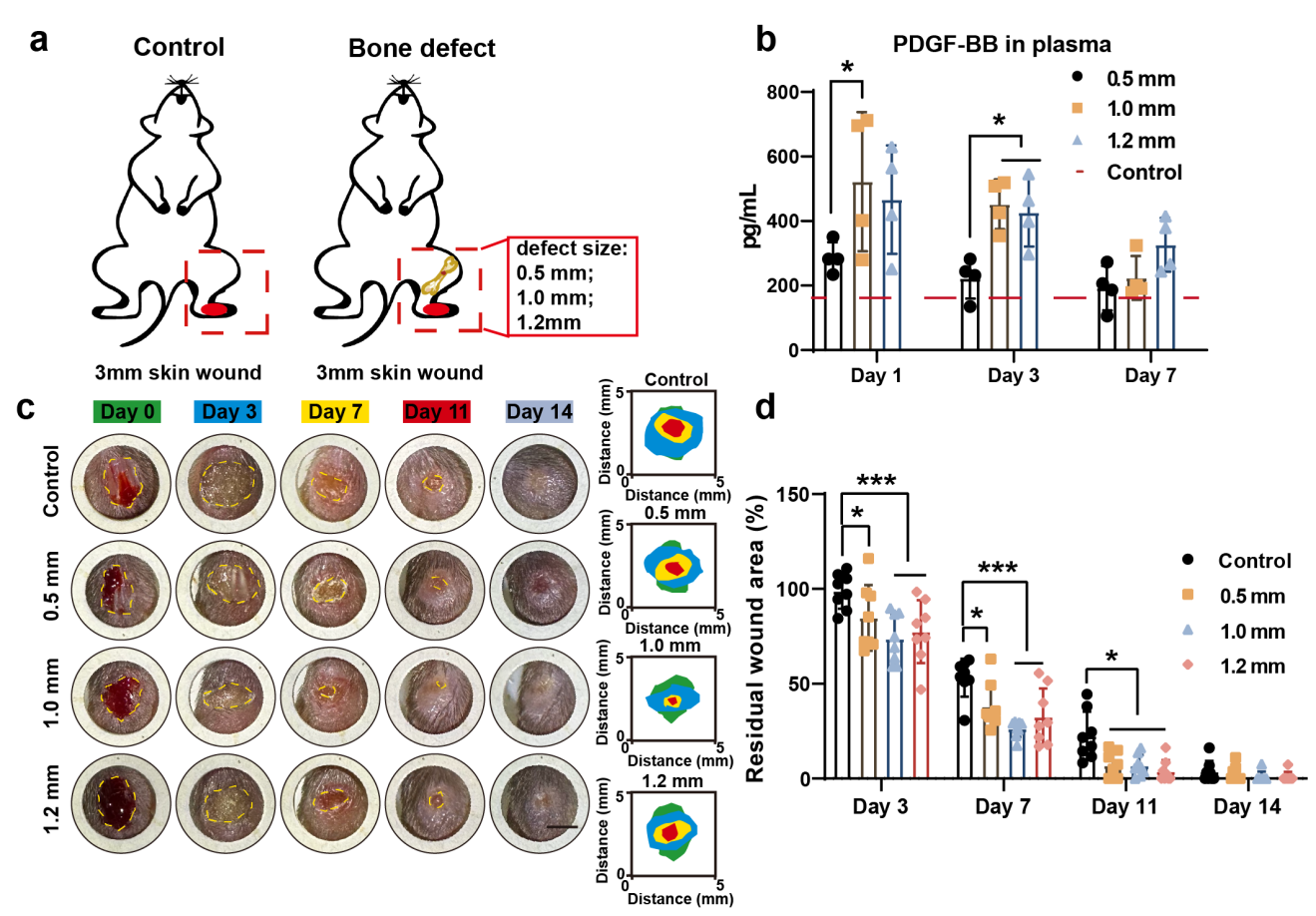


**Fig. S10.** **Evaluation of the effects of bone defects of varying sizes on diabetic foot wound healing.** (a) Schematic illustration of the surgical procedure. (b) Quantification of plasma PDGF-BB protein levels via ELISA. (n=4) (c) Representative images illustrating foot wound healing in the control group, the 0.5 mm group (bone defect size: 0.5 mm), the 1.0 mm group (bone defect size: 1.0 mm), and the 1.2 mm group (bone defect size: 1.2 mm). Yellow dashed line represents remaining wound contour. Scale bar, 2 mm. (d) Quantification of the percent of foot wound residual area at different times. (n = 8). Data are presented as mean ± SD and statistical significance was analyzed via two-way ANOVA with Tukey’s multiple comparison test for (b) and (d). P value: *P < 0.05, **P < 0.01, ***P < 0.001.


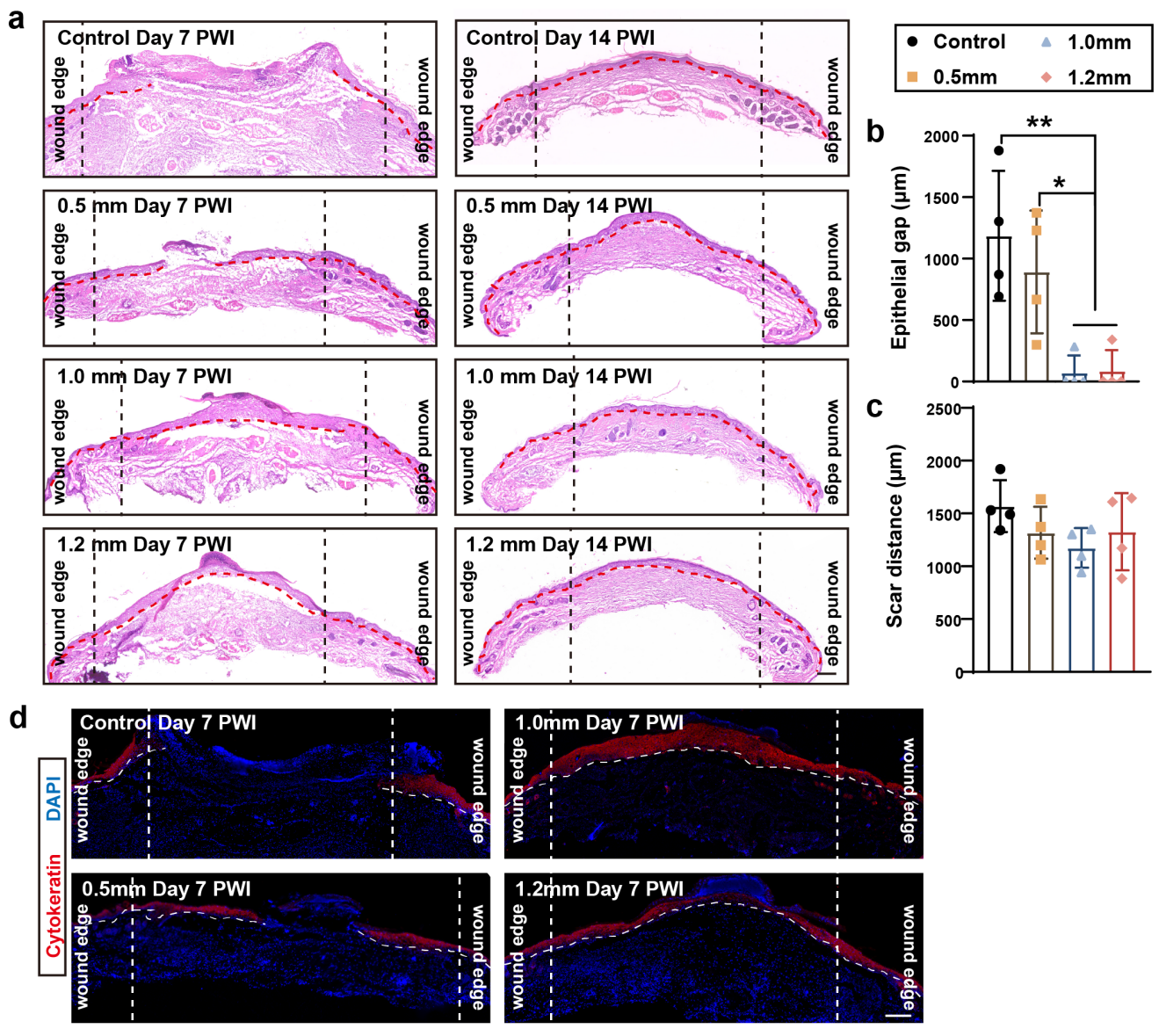


**Fig. S11.** **Histological analysis of diabetic foot wound healing under different sizes of bone defects.** (a) Representative H&E staining of foot wound tissue on day 7 and day 14. Scale bar, 200 μm. (b-c) Quantification of epidermis gap (b) on day 7 and scar distance (c) on day 14 according to HE staining images (n=4). (d) Sagittal sections of day 7 wound tissue immunolabeled for cytokeratin (red) and DAPI (blue). Scale bar, 200 μm. Data are presented as mean ± SD and statistical significance was analyzed via one-way ANOVA with Tukey’s multiple comparison test for (b) and (c). P value: *P < 0.05, **P < 0.01.


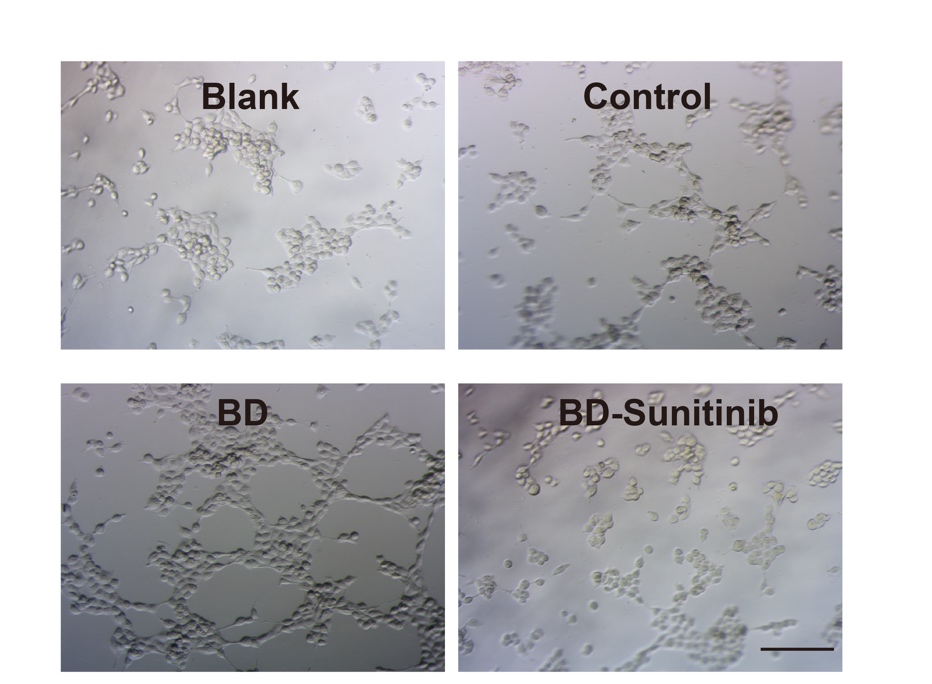


**Fig. S12. Representative tubular network formation images of HUVECs.** Cells treated with 2% FBS (Blank), 2% FBS+5% control plasma (Control), 2% FBS + 5% bone defect plasma (BD), and 2% FBS + 5% bone defect plasma + Sunitinib (BD+Sunitinib). Scale bars, 100 μm.


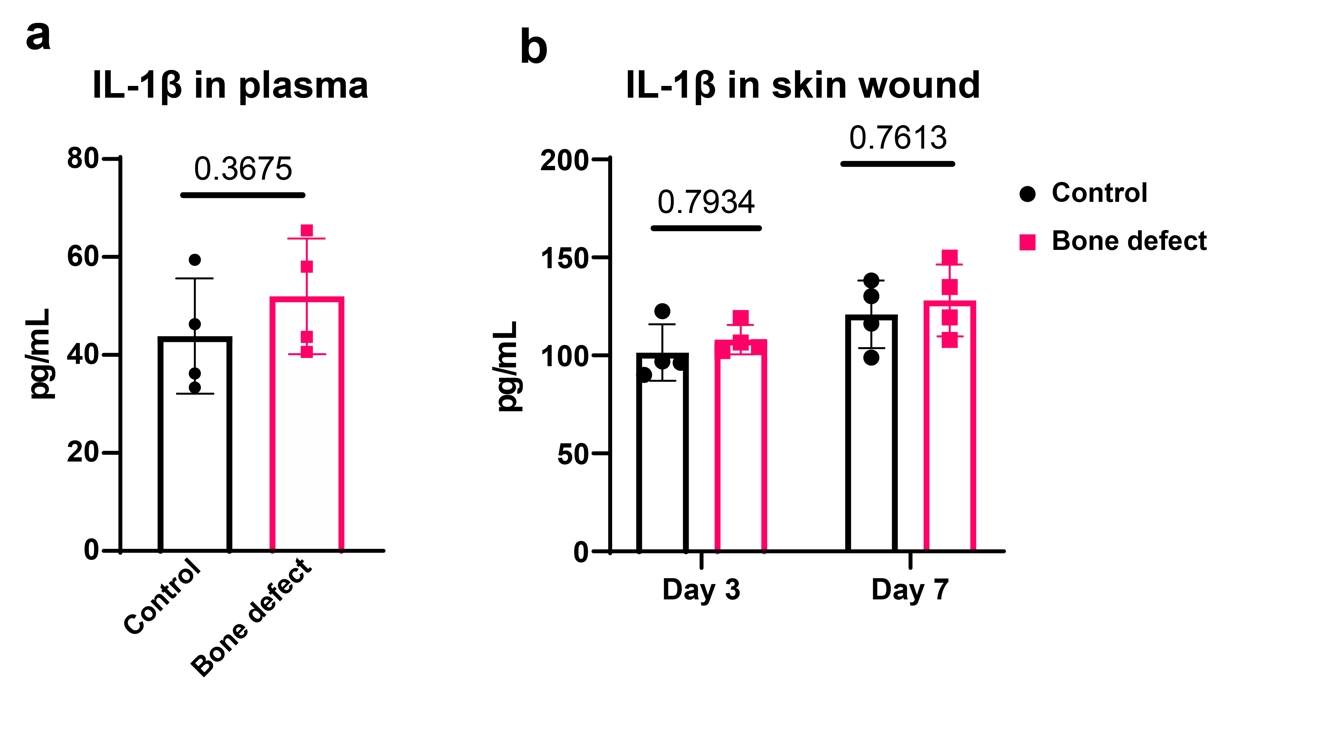


**Fig.** **S13. IL-1β protein expression.** IL-1β protein expression analysis by ELISA in plasma from control and bone defect diabetic mice (a), and in skin wound tissue (b) on days 3 and 7 post wounding (n = 4). Data are presented as mean ± SD and statistical significance was analyzed via unpaired two-tailed Student's t test for (a), two-way ANOVA with Tukey’s multiple comparison test for (b).


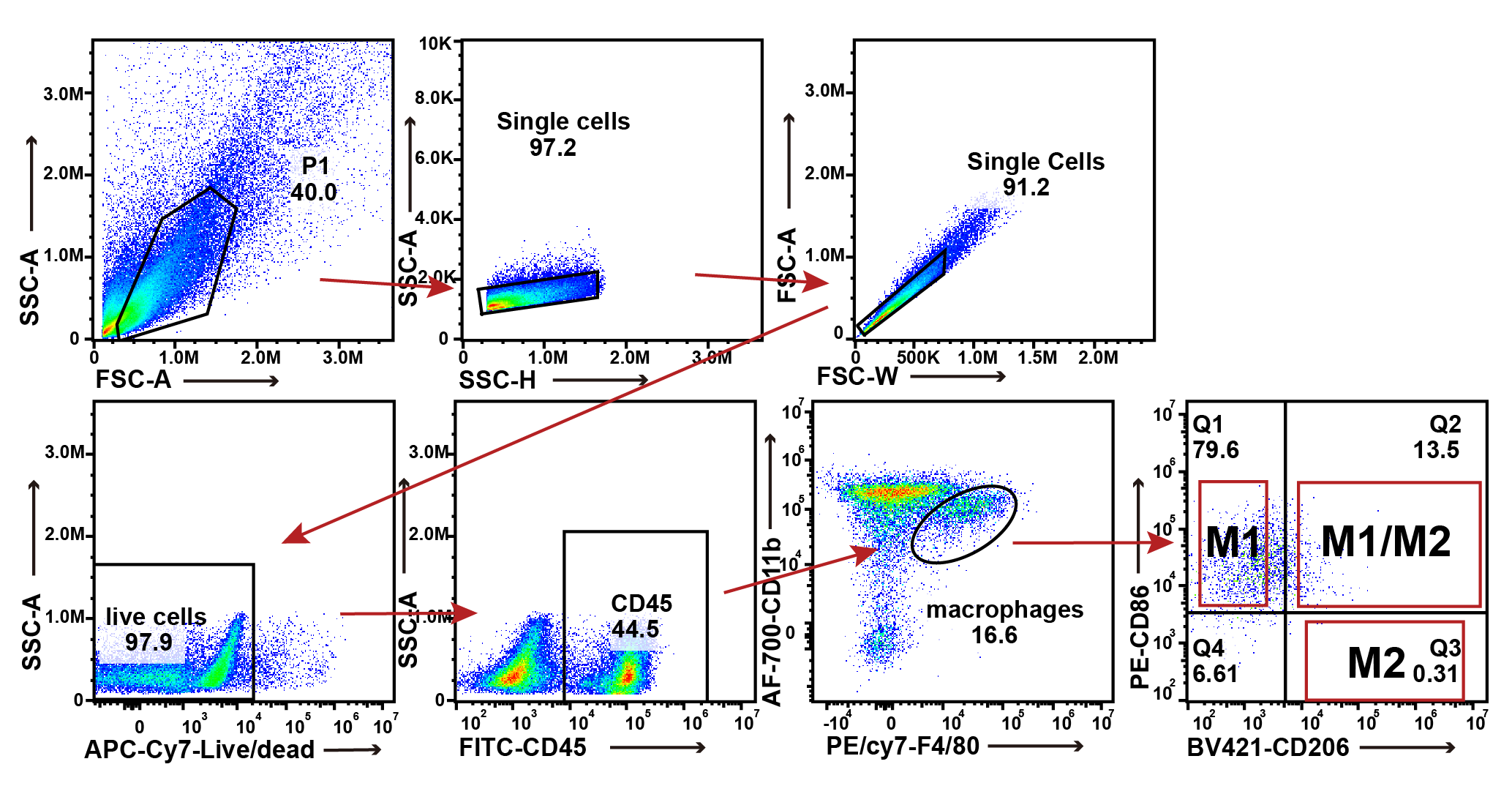


**Fig. S14. Gating strategies for macrophage analysis by flow cytometry in diabetic wounds.**


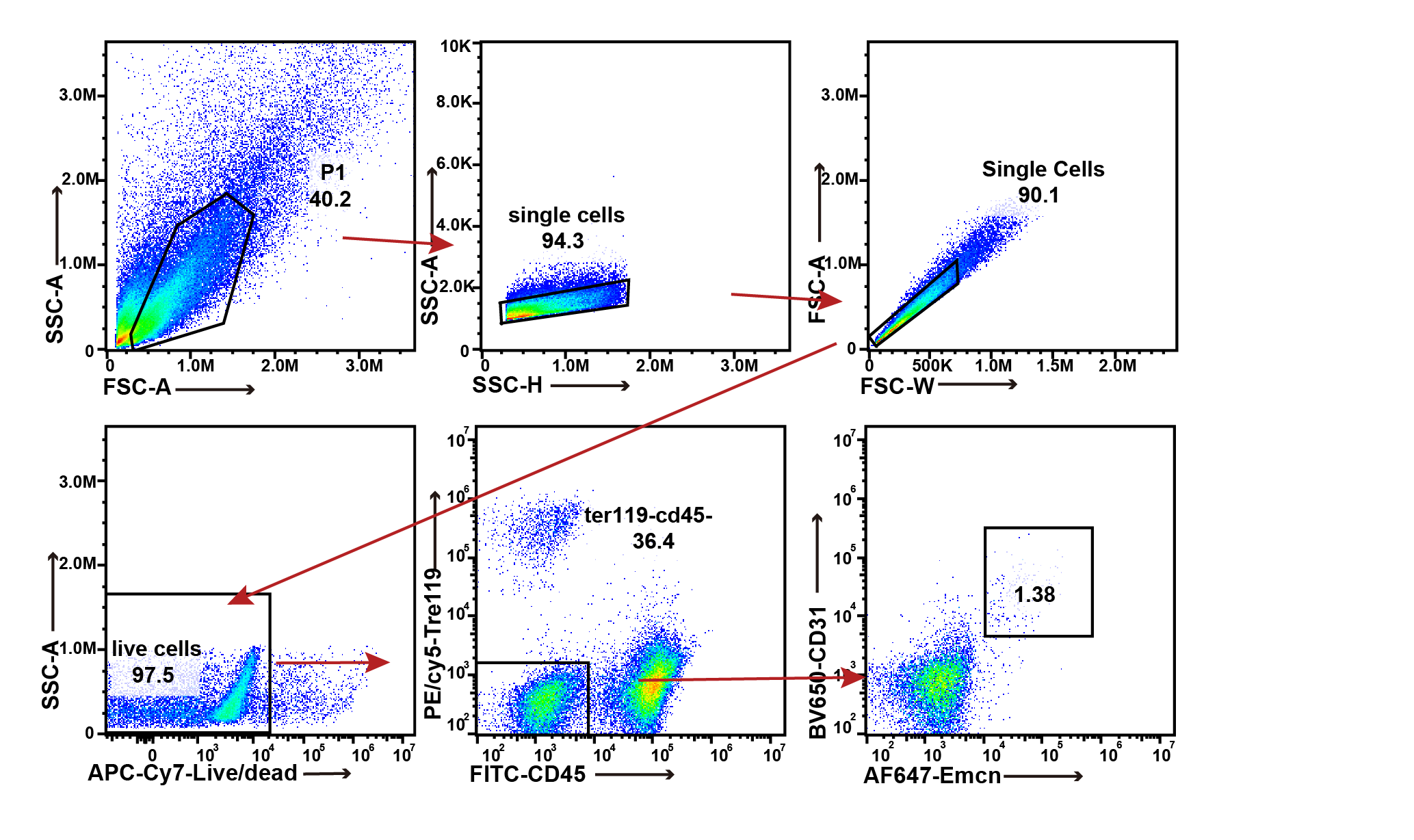


**Fig. S15. Gating strategies for angiogenesis analysis by flow cytometry in diabetic wounds.**

Table S1. PCR primers used in this study. Species (mouse).

| Gene | Forward | Reverse |
| --- | --- | --- |
| *Egf* | TTAACGGGACAGGACTAGAGAAA | AAGGAACTTAGAAGAACTCGGGA |
| *Il-6* | TCTATACCACTTCACAAGTCGGA | GAATTGCCATTGCACAACTCTTT |
| *Il-10* | AGCCTTATCGGAAATGATCCAGT | GGCCTTGTAGACACCTTGGT |
| *bfgf* | TGGTGACCACAAGCTGAATG | TCCCTTGATAGACACAACTCCTC |
| *Kgf* | TGGGCACTATATCTCTAGCTTGC | GGGTGCGACAGAACAGTCT |
| *Gapdh* | TGACCTCAACTACATGGTCTACA | CTTCCCATTCTCGGCCTTG |

Table S2. PCR primers used in this study. Species (human).

| Gene | Forward | Reverse |
| --- | --- | --- |
| *Efnbl* | GAGGCAGACAACACTGTCAAG | AGCTTCAGTAGTAGGACCGTC |
| *Mmp9* | TGTACCGCTATGGTTACACTCG | GGCAGGGACAGTTGCTTCT |
| *Mmp13* | CCAGACTTCACGATGGCATTG | GGCATCTCCTCCATAATTTGGC |
| *Gib2* | CATGTACGACGGCTTCTCCAT | AATCGCCCATCACAAAAGCTC |
| *Flrt2* | CGCTGCGACAGGAACTTTG | TGGAGGTAGAGTACGGTTACG |
| *Cdsn* | TCTCAGACCCCTGTAAGGACC | CGTTCCTGGCTTAAAAGATCCTG |
| *Itga5* | AGACATTCGATCCCTCTACAACT | TGGAGGTAGAGTACGGTTACG |
| *Pcdh7* | TGATCTTCGACGAGAACGAGT | AATCGCCCATCACAAAAGCTC |
| *Itga6* | GGCGGTGTTATGTCCTGAGTC | AATCGCCCATCACAAAAGCTC |
| *Pcdhga1* | GTGACTGAAAGAACACTGGACA | AATCGCCCATCACAAAAGCTC |
| *Gjb6* | CAAGAGGACTTCGTCTGCAAC | GTGGTTTCGTGCCTGTAGTAG |
| *Fn1* | CGGTGGCTGTCAGTCAAAG | AAACCTCGGCTTCCTCCATAA |
| *Lamc2* | CAAAGGTTCTCTTAGTGCTCGAT | CACTTGGAGTCTAGCAGTCTCT |
| *Lamb3* | GCAGCCTCACAACTACTACAG | CCAGGTCTTACCGAAGTCTGA |
| *Lama3* | CACCGGGATATTTCGGGAATC | AGCTGTCGCAATCATCACATT |
| *Cald1* | TCGACCCAACAATAACAGATGC | TCTCGTATCTTTCTTGGCGACT |
| *Nav2* | ATCAATGGCTGTCCGAAGAAC | TGGATGTTTATTCCCTTAGCTGC |
| *Tubb3* | GGCCAAGGGTCACTACACG | GCAGTCGCAGTTTTCACACTC |
| *Tubb6* | GGCCGGGATCTTACAGACAG | CCGCGTTGGAGTAATTGACAA |
| *Tubb2b* | GGCACGATGGATTCGGTTAGG | ACACGAAATTGTCTGGTCTGAAG |
| *Tpm1* | TTGAGAGTCGAGCCCAAAAAG | CATATTTGCGGTCGGCATCTT |
| *Tpm2* | AGTTTGCCGAGAGGTCTGTG | TCTCGTATCTTTCTTGGCGACT |
| *Myo5b* | CTGTGGTATCGTACTTGTTGCC | AACACGCCGTCTGAATTTCTT |
| *Myo1b* | CGGATGAAGCATACAGATCCC | CTGCCACATAGGACATGACAAG |
| *Myh9* | CTGTAGGCGGTGTCTGTGAT | CGGATGAAGCATACAGATCCC |
| *Fscn1* | CCAGGGTATGGACCTGTCTG | GTGTGGGTACGGAAGGCAC |
| *Fmnl2* | GCTATGAACCTACCTCCTGACA | AACACGCCGTCTGAATTTCTT |
| *Gapdh* | GGAGCGAGATCCCTCCAAAAT | GGCTGTTGTCATACTTCTCATGG |
